# RNA polymerases in strict endosymbiont bacteria with extreme genome reduction show distinct erosions that might result in limited and differential promoter recognition

**DOI:** 10.1371/journal.pone.0239350

**Published:** 2021-07-29

**Authors:** Cynthia Paola Rangel-Chávez, Edgardo Galán-Vásquez, Azucena Pescador-Tapia, Luis Delaye, Agustino Martínez-Antonio

**Affiliations:** 1 Biological Engineering Laboratory, Genetic Engineering Department, Center for Research and Advanced Studies of the National Polytechnic Institute, Irapuato Gto, México; 2 Departamento de Ingeniería de Sistemas Computacionales y Automatización, Instituto de Investigaciones en Matemáticas Aplicadas y en Sistemas, Universidad Nacional Autónoma de México, Coyoacán, Ciudad de México, CDMX, México; 3 Evolutionary Genomics Laboratory, Genetic Engineering Department, Center for Research and Advanced Studies of the National Polytechnic Institute, Irapuato Gto, México; Instituto de Biologia Molecular de Barcelona, SPAIN

## Abstract

Strict endosymbiont bacteria present high degree genome reduction, retain smaller proteins, and in some instances, lack complete functional domains compared to free-living counterparts. Until now, the mechanisms underlying these genetic reductions are not well understood. In this study, the conservation of RNA polymerases, the essential machinery for gene expression, is analyzed in endosymbiont bacteria with extreme genome reductions. We analyzed the RNA polymerase subunits to identify and define domains, subdomains, and specific amino acids involved in precise biological functions known in *Escherichia coli*. We also perform phylogenetic analysis and three-dimensional models over four lineages of endosymbiotic proteobacteria with the smallest genomes known to date: *Candidatus* Hodgkinia cicadicola, *Candidatus* Tremblaya phenacola, *Candidatus* Tremblaya Princeps, *Candidatus* Nasuia deltocephalinicola, and *Candidatus* Carsonella ruddii. We found that some Hodgkinia strains do not encode for the RNA polymerase α subunit. The rest encode genes for α, β, β’, and σ subunits to form the RNA polymerase. However, 16% shorter, on average, respect their orthologous in *E*. *coli*. In the α subunit, the amino-terminal domain is the most conserved. Regarding the β and β’ subunits, both the catalytic core and the assembly domains are the most conserved. However, they showed compensatory amino acid substitutions to adapt to changes in the σ subunit. Precisely, the most erosive diversity occurs within the σ subunit. We identified broad amino acid substitution even in those recognizing and binding to the -10-box promoter element. In an overall conceptual image, the RNA polymerase from *Candidatus* Nasuia conserved the highest similarity with *Escherichia coli* RNA polymerase and their σ^70^. It might be recognizing the two main promoter elements (-10 and -35) and the two promoter accessory elements (-10 extended and UP-element). In *Candidatus* Carsonella, the RNA polymerase could recognize all the promoter elements except the -10-box extended. In *Candidatus* Tremblaya and Hodgkinia, due to the α carboxyl-terminal domain absence, they might not recognize the UP-promoter element. We also identified the lack of the β flap-tip helix domain in most Hodgkinia’s that suggests the inability to bind the -35-box promoter element.

## Introduction

Until 2006, scientists thought that the minimum quantity of genes necessary to support life would be around 500. However, this view changed soon after when genomes from obligated endosymbiotic bacteria began to be published. Several of these genomes contained less than 500 genes, with extreme cases with less than 200 genes [1]. Some clues on how these minimal genomes managed to provide all the necessary functions to sustain life begin to emerge. For instance, transcriptome analysis of *Buchnera aphidicola* offers evidence that its genome has a limited ability to respond to environmental fluctuations [2]. Thus, gene expression in obligate endosymbiont is somehow stable and just active at basal levels. According to this, the density of promoter-like signals, characteristic of free-living bacteria, is not present in organisms exhibiting extreme genome reductions [3].

DNA transcription is an essential molecular process through which organisms decode genetic information into cellular functions [4]. RNA polymerase (RNAP) is the enzyme responsible for transcribing DNA to RNA. It consists of a multi-subunit protein complex present in all living organisms, from bacteria to eukaryotes [5]. In bacteria, the RNAP is responsible for synthesizing all RNAs, including messenger, ribosomal, transfer, and small RNAs. In free-living bacteria, the RNAP holoenzyme consists of six subunits (α_2_ββ’ωσ), encoded by five different genes (this includes two copies of the α subunit). The ordered assembly of these five proteins constitutes the holoenzyme with a molecular mass of around 400 kDa. Previous studies on *E*. *coli* found that the ω subunit is not essential for RNAP activity [6]. Later studies indicate that the ω subunit is absent in all endosymbionts analyzed herein [7]. The rest of the subunits are considered essential core components of RNAP (α_2_, β, and β’). They are well conserved in bacteria [5,8]. This RNAP core is catalytically active (transcribes DNA to RNA) but cannot initiate DNA transcription by itself. Instead, the RNAP core must bind to an additional subunit called the sigma factor (σ) to initiate transcription. The σ is responsible for recognizing and binding to gene promoters [8]. Once transcription begins, and after synthesizing a short fragment of RNA, the σ release and the RNAP core continue transcribing until it reaches a transcription terminator.

Since σ is responsible for binding and discriminating among gene promoters, it is common to find different types of σ. Those fall into two evolutionary families. One of these is called the σ^54^, which has a single member in free-living bacteria and is absent in endosymbionts. On the contrary, the σ^70^ family has several copies per genome (from 1 in endosymbionts to around 60 in free-living bacteria). One member of the σ^70^ family is also known as the "housekeeping σ factor," an essential gene present in all bacteria [9]. In *E*. *coli*, this housekeeping gene is precisely the σ^70^ or *rpoD* gene. One ortholog of this gene probably should encode the housekeeping σ in strict endosymbionts [7]. In *E*. *coli*, there are seven σ factors encoded in the *E*. *coli* genome, six of them correspond to the σ^70^ family (σ^70^, σ^38^, σ^32^, σ^28^, σ^24^ and σ^19^), and the remaining one corresponds to σ^54^. The architecture of transcription units and the consensus promoter sequences for each σ in *E*. *coli* was proposed previously [10].

### A brief functional description of the RNAP essential subunits in *E*. *coli*

#### The α subunit

In *E*. *coli*, *rpoA* encodes for the RNAP α subunit. This protein has a molecular weight of ~37 kDa with two folded domains (α carboxy and α amino-terminal domains, also known as α-CTD and α-NTD) connected by two flexible linkers [11]. This subunit performs three biological functions: i) it initiates the assembly of the RNAP complex through the interaction of its α-NTD with the β- and β’-subunits; ii) it participates in promoter recognition through the interaction of its α-CTD with the DNA UP promoter element, and iii) their α-CTD is also a target for the binding of many transcriptional activators. These transcription factors are bound in the following architectures: 1) where the α-CTD situates between a bound activator and the rest of the RNAP, 2) the α-CTD located upstream of a bound activator; 3) the α-CTD is flanking a bound activator, and 4) combinations of the above arrangements in the presence of multiple activators [12–18]. For instance, dimers of the cAMP receptor protein (dual transcriptional regulator CRP) interact with one of the two α-CTD [14,15]. In addition, the integration host factor (IHF) interacts with α-CTD to activate XylR [16]. Also, FIS activates transcriptionally through the α-CTD that is β’-associated, linked to the DNA on the promoter distal side of FIS [17].

#### The β and β’ subunits

*rpoB* and *rpoC* encode for the β and β’ subunits of RNAP in *E*. *coli*. The β subunit forms a pincer, called "clamp." The β’ subunit constitutes the other pincer. In between give place to a 27 Å-wide internal channel, where the catalytic site of the RNAP enzyme is located [18]. The β subunit binds to the α and β’ subunits through 24 and 73 amino acids distributed in the region between the amino acids 540 to 1340 [18]. The interaction of σ to the RNAP core is via the β subunit also, through their β flap-tip helix domain [19].

In the β’ subunit, the amino acids present in the region from 917 to 1361 are essential for their interaction with the β subunit. And their β’ coiled-coil domain for their interaction with the σ factor. Additionally, in the β’ amino-terminal domain, several functional amino acids, typical of Zinc fingers, form the RNAP catalytic site and stabilizes the RNAP-DNA complex. The active site is composed of around 500 amino acids retaining two Mg^2+^ ions. However, only thirteen amino acid residues are the most conserved conforming to the catalytic site containing three aspartic amino acids. On the other hand, in the β’ CTD domain, three polar residues and the G-loop domain conform to the cavity where the DNA fits and contact the RNAP [20].

Once assembled the RNAP core (α_2_ββ’), σ joins it by interacting with the β and β’subunits. First, σ binds to β’ through the β’ coiled-coil domain. Such binding places σ to contact the -10-box element to form the open promoter complex [21]. Likewise, the β subunit binds to the σ through the β flap-tip helix domain at the σ_4_ region (see below). This contact gives σ the capacity to adapt to variation in nucleotide distances between the -10 and the -35-box promoter elements [19].

#### The σ subunit

The σ factor is not a permanent component of the RNAP core but a transitory-associated subunit. The σ factor is necessary for promoter recognition and transcription initiation. In *E*. *coli*, σ^70^ consists of a protein with four helical domains (σ_1_, σ_2_, σ_3_, and σ_4_). Each of them interacts with different promoter elements and domains of the different RNAP core subunits. The σ_1_ domain prevents σ^70^ from interacting with the DNA strand without a complete RNAP core [22,23]. The σ_2_ domain is the most conserved of the σ^70^ family and consist of four subdomains: σ_2.1_, σ_2.2_, σ_2.3,_ and σ_2.4_. The σ_2.1_ and σ_2.2_ subdomains are involved in the binding to the RNAP core [24]. The σ_2.2_ subdomain contains sites for binding the β’ coiled-coil domain. In contrast, the σ_2.3_ subdomain participates in DNA melting. It has seven conserved aromatic amino acids, whose replacement results in defects in DNA melting [25,26]. The specific recognition of the -10-box promoter element is a function of the σ_2.4_ subdomain. The amino acids involved in DNA melting and binding of the -10-box promoter element interact on the same DNA helices’ faces. Deletion analyses have determined that the σ_2_ domain seems necessary for the correct functioning of the RNAP [26,27]. The σ_3_ region interacts with the -10-box extended promoter element. It stabilizes the short nascent DNA-RNA hybrids during the early stages of gene transcription [28] because of their interaction with the 5’-triphosphate of the nascent RNA [29]. Finally, the σ_4_ domain is formed by two subdomains, σ_4.1_ and σ_4.2_. The σ_4.1_ subdomain interacts with the β flap-tip helix of the β subunit to allow their correct binding to the -35-box promoter element. More specifically, the amino acids R518 and R516 in σ_4.2_ recognize the guanine and cytosine at the -34 and -32 nucleotide positions concerning the transcription start (+1) [27]. Additionally, the σ_4.1_ subdomain is a point of contact with transcriptional activators that bind upstream of the -35-box promoter element. One study reports that the binding of the σ_4.1_ subdomain of σ^38^ with the β flap-tip helix domain is more potent than with this subdomain in σ^70^ [30].

### Endosymbiotic bacteria

Endosymbiotic bacteria live inside other organisms (usually eukaryotes). These show genomic features resulting from several million years of co-evolution [31]. Strict endosymbiotic bacteria cannot outside their host and have lost most of their genes and large fragments of amino acids in their remaining proteins [7,10]. The comparison of *E*. *coli* genome, with approximately 4.5 thousand genes to the endosymbiotic bacterium *Candidatus* Carsonella ruddii, which has retained just around 150 genes, exhibits this dramatic process [32,33]. In addition, almost all genes found in *Candidatus* Carsonella ruddii are considerably shorter than their free-living orthologues.

Furthermore, genes encoding proteins with multiple domains in obligate endosymbionts commonly have lost some regions or complete domains. These, in some cases, are essential for their activity in free-living bacteria [34,35]. For instance, in previous work, we determined that obligate endosymbionts had lost all the transcription factors that interact with the promoters and RNAP to activate or inhibit gene expression [36]. Additionally, partial loss of the α and σ subunits is present in *Candidatus* Hodgkinia sp. and *Candidatus* Carsonella ruddii [35,37]. Both are considered essential components of the RNAP in free-living bacteria.

Gene erosion of the transcriptional machinery in endosymbionts with highly reduced genomes raises the critical question of how gene transcription could be happening in these bacteria. Here, we address this question from the perspective of comparative genomics. With this purpose, we investigated the RNAP subunits in four bacterial lineages of obligate endosymbionts: *Candidatus* Hodgkinia cicadicola, *Candidatus* Tremblaya phenacola, and princeps, *Candidatus* Nasuia deltocephalinicola, and *Candidatus* Carsonella ruddii, which exhibit extreme genome-reduction, within the proteobacteria phylum.

## Materials and methods

### Genome data of selected endosymbiont

To recover sequenced genomes, we use the NCBI database (http://www.ncbi.nlm.nih.gov/). In this database, we identified 37 endosymbionts with extreme genome reduction. All these pertain to the four lineages of proteobacteria. *Candidatus* Hodgkinia cicadicola belongs to α-proteobacteria. *Candidatus* Tremblaya phenacola; *Candidatus* Tremblaya princeps; and *Candidatus* Nasuia deltocephalinicola, which belong to β-proteobacteria. Moreover, *Candidatus* Carsonella ruddii, which belongs to γ-proteobacteria. The genomes of these bacteria are completely sequenced. Further characteristics of these genomes are in the supplementary material S1 Table.

### Amino acid alignment of the RNAP subunits in strict endosymbiotic bacteria and their comparison to *E*. *coli*

We compared the amino acid sequences for each RNAP subunit against their corresponding *E*. *coli* orthologous. We use the T-Coffee program in a multiple sequence alignment with standard parameters [38]. For simplicity, the amino acid positions will from now on be referred to their location in the corresponding *E*. *coli* protein or subunit, accompanied by the abbreviation "ECO." With this alignment, we define domains, subdomains, and relevant amino acids. We also utilized the Pfam database [39], the proteins superfamily classification [40], and the NCBI’s conserved domains [41]. In addition, we did bibliographic research to gather relevant information regarding the sites and amino acid regions for each of the RNAP [11–13,18–21,23–28,30,42–45].

### Structural analysis of the RNAP subunits and inference of their 3-D structural-functional models

We first recreated 3-D structural models for each RNAP subunit using the I-TASSER server with standard parameters [46]. I-TASSER generates 3-D models for a given sequence by collecting high-score structural templates from PDB (Protein Data Bank) with full-length atomic models constructed by iterative template-based fragment assembly simulations. The structural model of *Candidatus* Hodgkinia TETUND2 and Dsem strains was obtained from their homologous proteins in *E*. *coli* by using the multiple threading alignments.

Then we superpose the resulting 3-D models with the crystal structure of the RNAP ECO (4YLP) [47]. Graphic representations of each structure were prepared with the PyMOL Molecular Graphic System software version 1.3 [48]. For the interaction of RNAP with the promoter sequence, we used the predicted models of the RNAP subunits obtained by I-TASSER for *Candidatus* Hodgkinia Dsem and TETUND2. We did a structural alignment with the homologous structure of the holoenzyme RNAP of *E*. *coli* (ECO 4YLP) in PyMOL.

We find that *Candidatus* Hodgkinia TETUND2 has lost a fragment of the α-NTD involved in dimer formation. To investigate the possible dimer association of α subunit monomers in this bacterium, we used ClusPro v.2.0 [49–52]. This tool is an automatic protein docking tool based on CAPRI (Critical Assessment of Predicted Interactions) [50,53]. As a result, we get three models of α dimer subunits with different cluster sizes. We map sites under putative positive selection with these models and evaluate free-energy changes in the protein-protein interactions. With this strategy, we recreated single mutations along with the amino acids positively selected using the BindProfX server [54]. Following this, we exchanged the putative positive selected amino acid on the dimers predicted in the different strains of Hodgkinia. Then we determined the changes in protein binding affinity. The binding affinities between each pair of proteins were measured as Gibbs free-energy change ΔG = G (complex) -G (monomers). When two monomers form a complex, the more negative a ΔG is, the more stable the complex results. Finally, we calculate the effect of mutations on binding affinity by the differences in free-energy changes between the mutant and the wild type ΔΔG_wt->mut_ = ΔGmut-ΔGwt. The criteria to consider a strongly favorable mutation was to have ΔΔG≤-1kcal/mol.

### Natural selection analysis of the RNAP subunits from endosymbionts

To understand the putative mechanisms of RNAP subunits’ molecular evolution, first, we need to know the selective pressure acting on each of the subunits of this protein. For this purpose, we estimated the D_N_/D_S_ ratio (ω) on the protein-coding sequences studied here. The ratio considers non-synonymous substitutions per non-synonymous sites (D_N_), divided by the number of synonymous substitutions per synonymous sites (D_S_). The D_N_/D_S_ ratio can result in three evolutionary processes: i) if D_N_/D_S_ < 1, we infer purifying selection; ii) if D_N_/D_S_ > 1, then we infer positive selection; and iii) if the D_N_/D_S_ = 1, it indicates a neutral evolution [55]. We perform these selection analyses to RNAP subunits in each group of bacteria.

Natural selection was inferred with CodeML from PAML v.4.6 package [56]. This software requires codons alignment. For those, we used PAL2NAL v.2.1.0 program [57]. To graph the phylogenetic trees, we use PhyML [58]. We previously aligned the set of amino acid sequences of each subunit with T-Coffee [38]. We used Gblocks to recover the informative codons [59]. To identify specific genes and amino acids under positive selection, we use branch and branch-site models. In the case of a branch, we use three models: "M0" one-ratio model (D_N_/D_S0_), free model (D_N_/D_S1_), and two-ratio model D_N_/D_S2_. The D_N_/D_S0_ model assumes the same D_N_/D_S_ ratio for all the branches. The D_N_/D_S1_ assumes an independent D_N_/D_S_ for each branch. The D_N_/D_S2_ assumes that the branch of interest (foreground branch) has a D_N_/D_S2_ ratio different than the background ratio [60].

The level of significance for the Likelihood Ratio Test (LRT) was estimated using the *x*^*2*^ distribution with degrees of freedom (*df*). These degrees of freedom are equal to the difference in the number of parameters between the models. The statistic considers twice the difference of log-likelihood between the models (2ΔlnL = 2[lnL_1_-lnL_0_]): where L_1_ and L_0_ are the likelihoods for the alternative and null models [61]. We compared one-ratio and free-ratio models to know whether D_N_/D_S_ were different among the lineages. In contrast, we examine whether the lineage of interest has a different ratio than the other lineages with one-ratio and two-ratio models.

We approached a model where the D_N_/D_S_ ratio was 1, 0.2, and 1.2 for the foreground branch to detect positive or negative selection in specific lineages. First, we compared D_N_/D_S2_ against the D_N_/D_S = 1_, where the null hypothesis is that models are not significantly different. Suppose the null hypothesis is rejected (p<0.05) and the two-ratio model is greater than 1. In that case, it indicates the possibility of positive selection in the foreground. Otherwise, if the two-ratio model estimate is smaller than 1, it is indicative of negative selection.

On the other hand, if the null hypothesis is accepted, it is evidence that the foreground branch is under neutral evolution. Additionally, we compared D_N_/D_S2_ against the D_N_/D_S = 0.2_; the null hypothesis is that models are not significantly different. For example, suppose the null hypothesis is (p<0.05), and the two-ratio model estimate is more significant than D_N_/D_S_ = 0.2. It indicates a weaker negative selection, while a value smaller than D_N_/D_S_ = 0.2 indicates a more substantial negative selection. Finally, when D_N_/D_S2_ is greater than one and is significantly different from D_N_/D_S = 1_, it means positive selection. To get more evidence about the likely positive selection, we compared D_N_/D_S2_ against D_N_/D_S = 1.2_; the null hypothesis was a non-significant difference between D_N_/D_S2_ and D_N_/D_S = 1.2_. Therefore, accepting the null hypothesis indicates that the foreground branch is possibly under positive selection. At the same time, a rejection means that the foreground branch might be subjected to a relaxed selection.

We performed a branch-site test for positive selection to identify individual codons under positive selection along specific branches [62]. In these models, positive selection was allowed on a particular "foreground" branch. We compared the LRTs (*df* = 1) against null models that assume no positive selection is happening. This test results in four classes of sites: 0, 1, 2a, and 2b. For the site classes 0 and 1, all codons are under purifying selection (0< D_N_/D_S0_<1) and neutral evolution (D_N_/D_S1_ = 1) for all branches. For sites in classes 2a and 2b, positive selection is allowed on the foreground branches (D_N_/D_S2_>1). For the rest, the "background branches" are under purifying selection (0<D_N_/D_S0_<1) and neutral evolution (D_N_/D_S1_ = 1). For the null model, D_N_/D_S2_ is 1. We test all the RNAP subunits in each endosymbiont in these ways. Each branch is considered as the foreground to reconstruct the phylogenies. We compared the two models using LRT. The calculus of significance between the models was twice the log-likelihood difference following an *x*^2^ distribution. With a *df* number equal to the difference of the number of parameters between the models. Positively selected amino acids were identified based on Empirical Bayes, and posterior probabilities were employed in CodeML [63]. We did not test the *Nasuia* RNAP subunits because there were only three sequenced strains. However, CodeML requires at least 4 to get reliable results.

## Results

**Fig 1:**
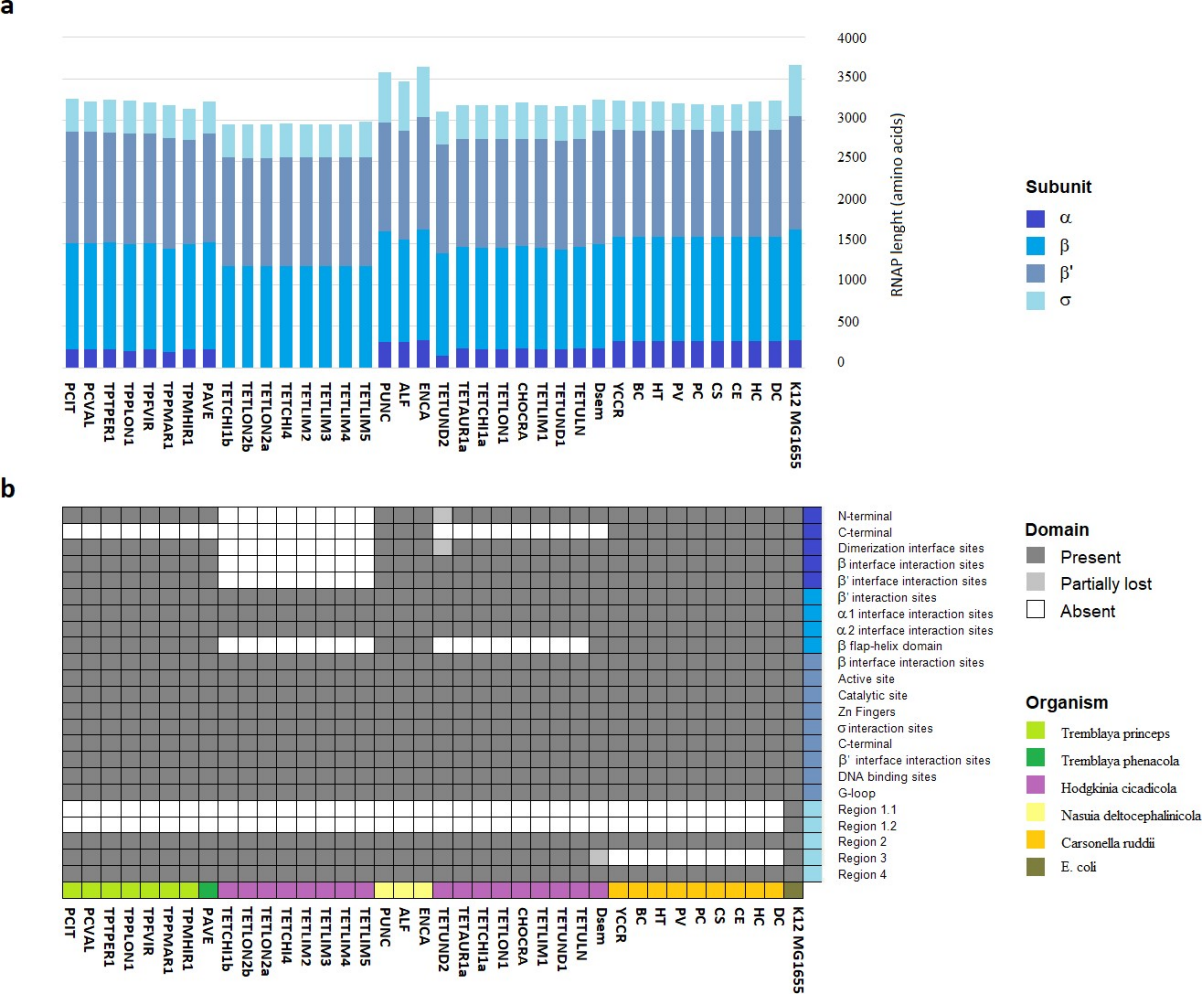
Conservation of RNAP subunits in endosymbiotic bacteria showing extremely reduced genomes. **a**) The bars represent the size (in amino acids) of RNAP subunits found in each endosymbiont; the different blue colors represent the relative size contribution of each RNAP subunits. **b**) Colors squares represents the degree conservation of functional domains in the RNAP subunits of *Candidatus* T*remblaya princeps* (lime green), *Candidatus* Tremblaya phenacola (dark green), *Candidatus* Hodgkinia cicadicola (magenta), *Candidatus* Nasuia deltocephalinicola (yellow), *Candidatus* Carsonella ruddii (orange), and *E*. *coli* (olive green).

To study the evolution of RNAP subunits in genomes exhibiting extreme reduction, first, we identified orthologous to the *E*. *coli* RNAP in the 37 obligate endosymbiotic bacteria (Fig 1A and S1 Table). The initial genomes included nine strains of *Candidatus* Carsonella ruddii, seventeen of *Candidatus* Hodgkinia cicadicola, only one of *Candidatus* Tremblaya phenacola, seven of *Candidatus* Tremblaya princeps, and three of Nasuia deltocephalinicola.

Nine of the seventeen Hodgkinia strains (52%) lack α subunits but were considered in the remainder analyses for the rest of the RNAP subunits. The rest (28 genomes) conserve orthologous genes for each of the α, β, β’ subunits, as well as a single gene coding for the σ factor. The total amino acid sequences encoding for each of the RNAP subunits exhibit a reduction of 16% on average, compared to those in *E*. *coli*. Also, we observed that these genes had lost DNA regions encoding important functional protein domains in *E*. *coli*. In some cases, with the loss of total domains (see below) (Fig 1B). In the following sections, we describe the structure and amino acid diversity found in each of the RNAP subunits of these endosymbiotic bacteria.

### The α-NTD is more conserved than α-CTD in the α subunit

**Fig 2:**
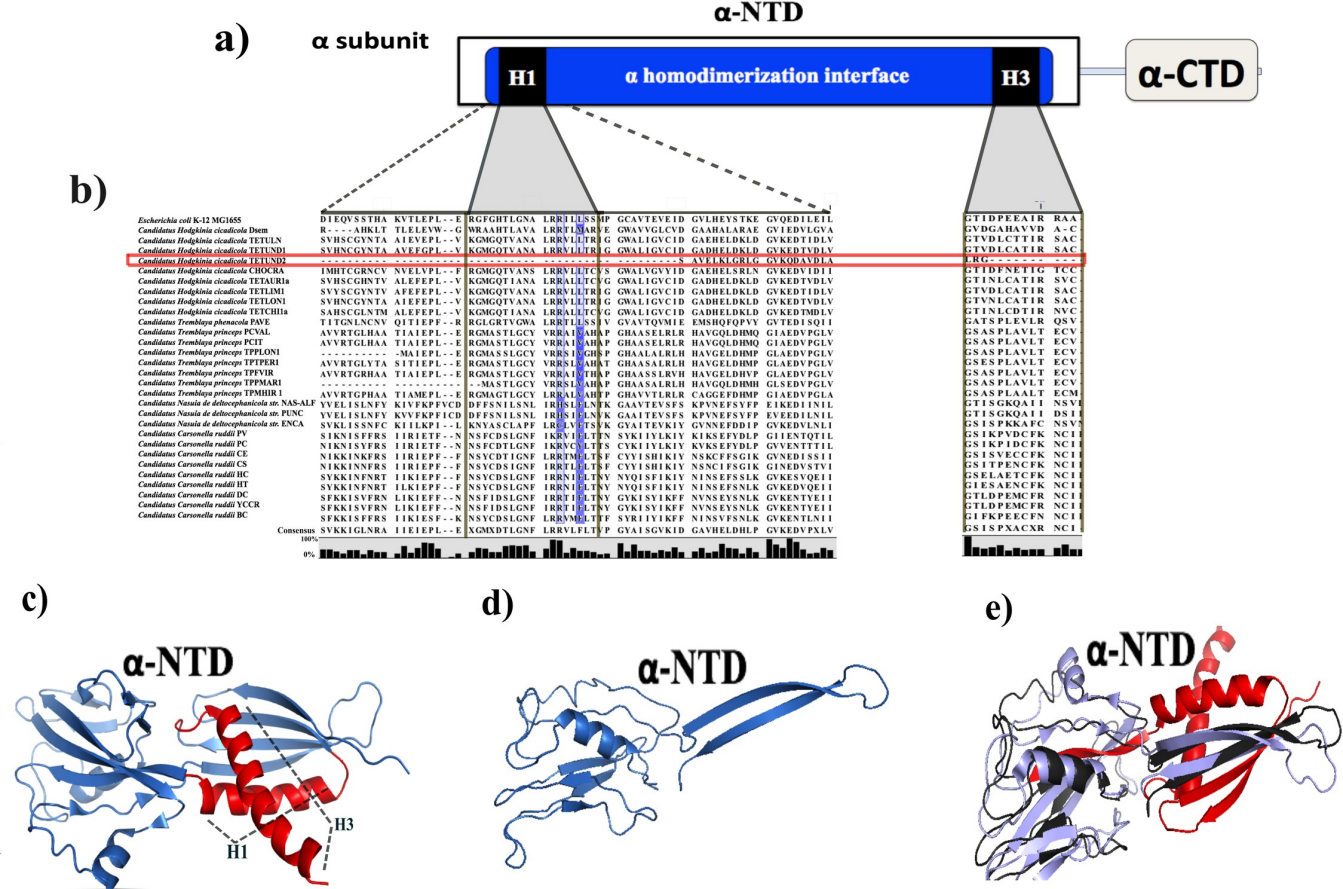
Domains and amino acid conservation in the α-subunit. **a)** The white and grey rectangle represents the α-NTD and α-CTD. Dark blue represents the α subunit dimerization region. The regions to form the H1 and H3 helices are shown in black rectangles. **b)** The amino acid alignment of the α subunits shows the conserved, functional amino acids involved in the dimer formation and the H1 and H3 α-helices. Therefore, darker backgrounds are showing those amino acids that differ from (*E*. *coli)*. **c)** 3-D *E*. *coli* α-NTD (4YLP) crystal structure shows red regions absent in *Candidatus* Hodgkinia TETUND2. **d)** The predicted 3-D model obtained for the α subunit of Hodgkinia TETUND2. **e)** Structural comparison between the *E*. *coli* α-NTD (4YLP) crystal structure (violet) and the predicted 3-D model obtained for the α subunit of Hodgkinia TETUND2 (black). In red, it shows the α-NTD regions absent in the α subunit of Hodgkinia TETUND2.

In Carsonella and Nasuia, their α subunits conserved all the functional domains known in *E*. *coli*. At the same time, Hodgkinia and Tremblaya mostly retain the α-NTD (for self-homodimerization and the interaction with β and β’ subunits) (Fig 2A and S1 Fig). *In vitro* and *in vivo* experiments revealed that the α-NTD is essential for RNAP to get basal transcription [64,65]. In addition, studies have shown that the α-CTD is not necessary for RNAP assembly and basal transcription. However, the α subunit requires α-CTD to interact with the UP-promoter elements and transcriptional activators in *E*. *coli* [66,67]. The loss of the α-CTD is also present in *Parcubacteria*. These are ectosymbiont bacteria that live in mixed groups [68]. Lack of α-CTD is also present in microalgae chloroplasts [69].

Two *Tremblaya princeps* strains, TPPLON1 and TPPMAR1, showed incomplete α-NTD. However, they preserved the essential regions for homodimerization and those for interaction with the β and β’ subunits. A particular case is *Candidatus* Hodgkinia cicadicola TETUND 2. This strain conserves just the region of α-NTD for interaction with the β’ subunit and only some amino acids for homodimerization (Fig 2B, red line). It is necessary to mention that the α-NTD consists of two well-conserved subdomains. Subdomain 1 contains two orthogonal α-helices (H1 and H3) called the homodimerization region (Fig 2C). Subdomain 2 includes the interfaces for interactions with the β and β’ subunits [41–43]. The first step towards RNAP core formation is the homodimerization between two monomers of α subunits. This dimer proceeds by the interaction of H1 and H3 helices in the subdomains 1 of each monomer. The 3-D predicted model of this subunit of *Candidatus* Hodgkinia TETUND2 shows that it does not conserve the H1 and just some amino acids of H3 helices and other motifs necessary to form the dimers interface (Fig 2D and 2E). Based on *E*. *coli*, we cannot infer if homodimerization is happening in the α subunits of *Candidatus* Hodgkinia TETUND2. The *rpoA* is not the unique case of genes losing a significant fragment in *Candidatus* Hodgkinia *cicadicola* TETUND 2. The DNA gene that encodes the ε subunit of DNA polymerase III has also lost large fragments. In such a way that it is no more considered a functional protein [33]. Finally, rest the nine Hodgkinias strains that lack a complete α subunit. The authors who reported these genomes say these bacteria were the most prevailing among several coexisting strains, all with fragmented genomes [66]. These nine bacteria conserve the other RNAP subunits, but it is difficult to infer if their RNAP remains functional.

### Strict endosymbionts conserve the β and β’ subunits except for the β flap-tip helix domain in Hodgkinias

**Fig 3:**
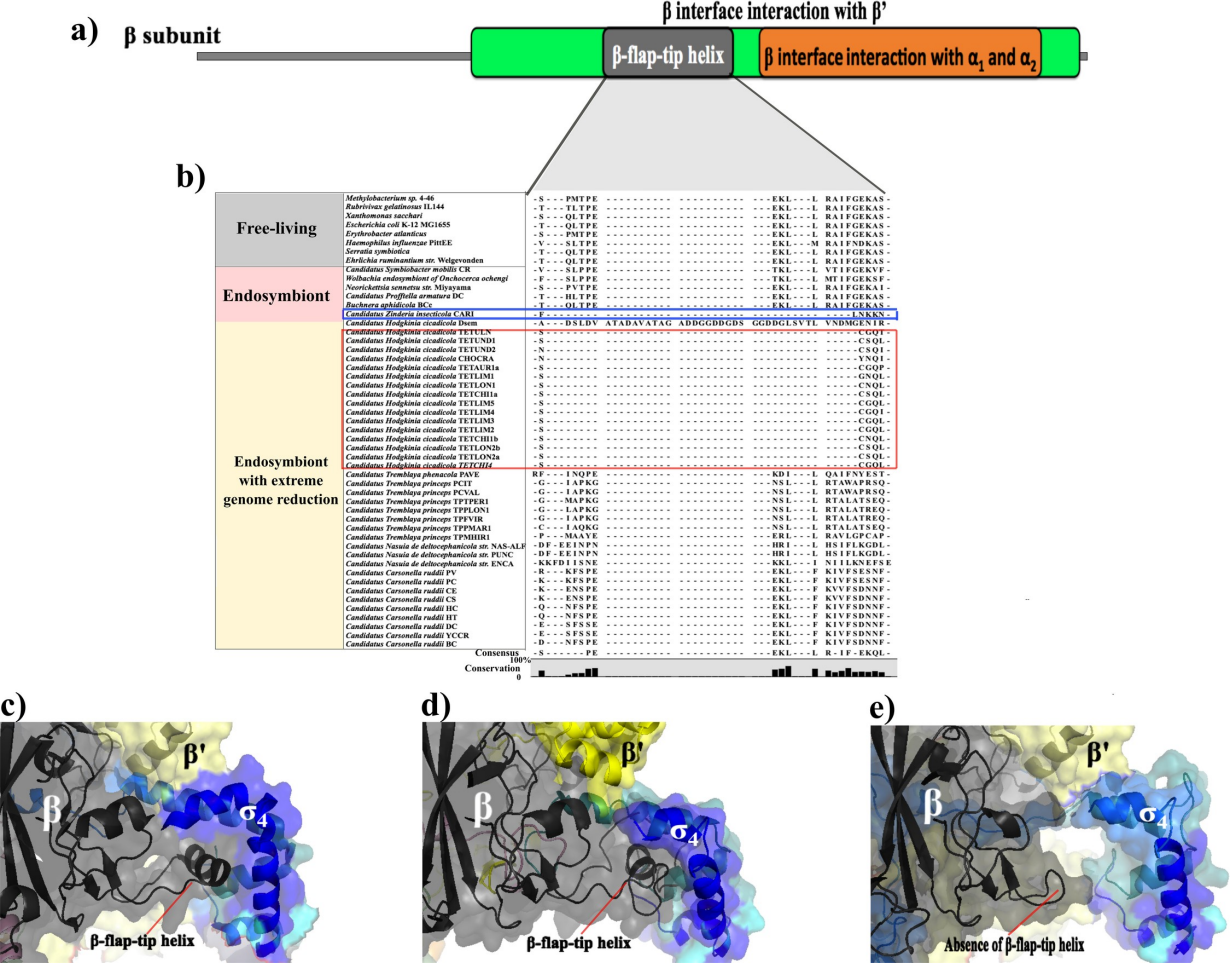
Amino acids sequence conservation in the β subunit. **a)** The upper figure represents the structural domains of the β subunit in *E*. *coli*. Besides the β flap-tip helix domain (grey region), the interaction regions with the two α and the β’ subunits (orange and pea-green). **b)** Amino acid alignment shows that most Hodgkinias lost the β flap-tip helix domain (red box). Also observed in *Candidatus* Zinderia cicadicola (blue box). **c)** Crystallographic structure of the *E*. *coli* β flap-tip helix and their interaction with σ_4_ (4YLP grey and blue). **d)** Predicted 3-D model for the β and σ subunits of Hodgkinia Dsem shows the interaction between the β flap-tip helix (grey) and the σ_4_ subdomain (blue). **e)** The predicted model for the β and σ subunits of Hodgkinia TETUND2 shows that, like the rest of Hodgkinias, the β flap-tip helix (grey) is not present. As a result, the interaction between the β subunit and the σ_4_ subdomain (blue) might be deficient.

**Fig 4:**
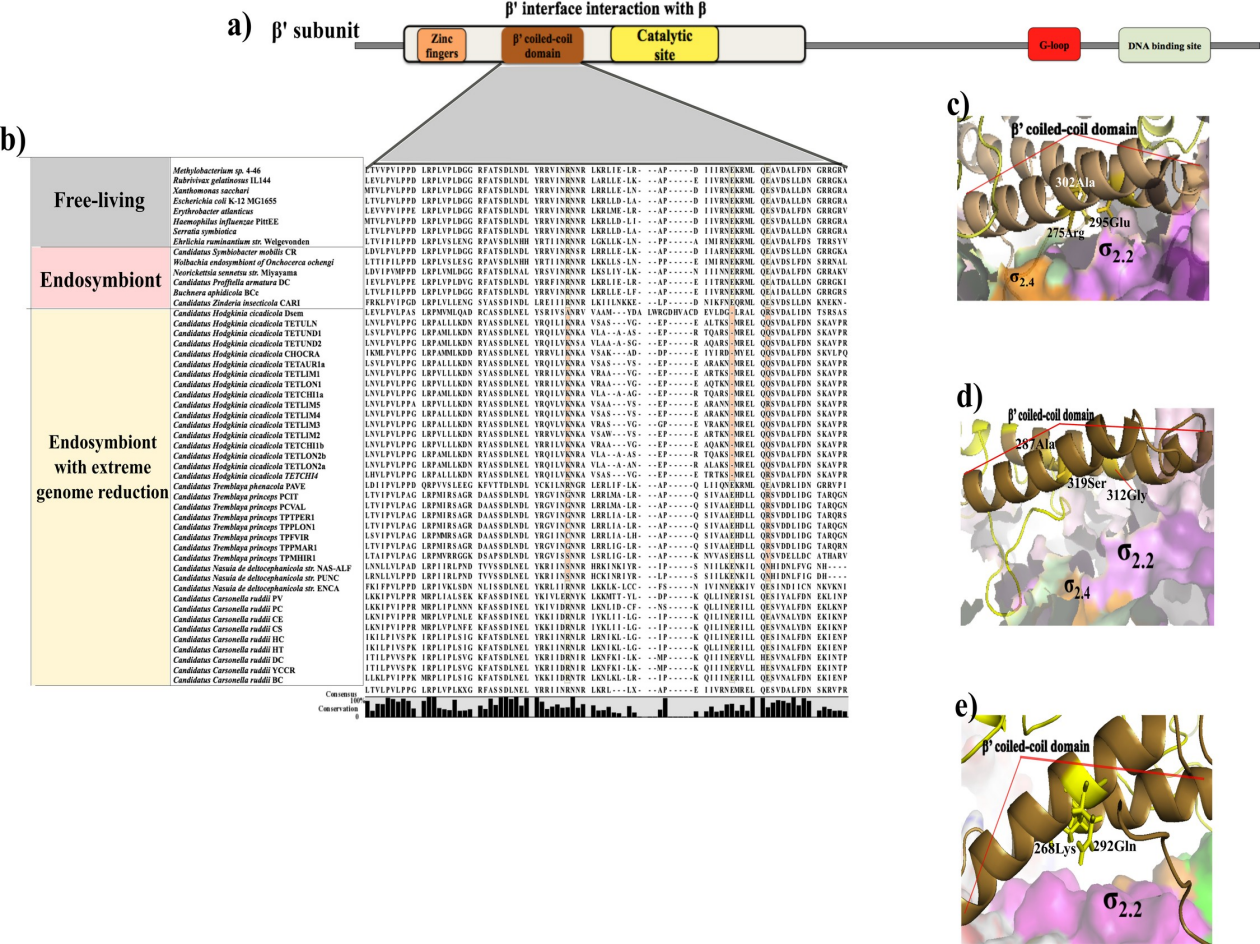
Amino acids sequence conservation in the β’ subunit. **a)** The figure in the upper part represents the position of functional domains in the β’ subunit, such as the β’ coiled-coil (brown), Zinc fingers (orange), the catalytic site (yellow), the G-loop (red), and the DNA-binding site (olive). The figure also shows the β’ interaction interface with the β subunit (beige) **b)** The alignment of β’ subunits shows substitution in the essential amino acids ECO: R275, E295, and A302 in the β’ coiled-coil domain. Darker backgrounds show those functional residues that differ from the reference *E*. *coli*. **c**) The crystallographic structure of the β’ coiled-coil domain of *E*. *coli* (brown). It shows the amino acid residues (in yellow) involved in the interaction with the σ_2.2_ domain (magenta). **d)** Predicted 3-D model for the β’ subunit in *Candidatus* Hodgkinia Dsem shows that the β’ coiled-coil domain could occur (brown). The amino acids A287, G312, and S319 (yellow) are involved in the interaction with the σ_2.2_ domain (magenta). **e)** Predicted 3D model for the β’ subunit in Hodgkinia TETUND2 shows that the β’ coiled-coil domain formation could also occur (brown region). The amino acids K268 and Q292 (yellow) could interact with the σ_2.2_ domain (magenta).

We identified that the β- and β’-subunits are the most conserved among all the RNAP subunits in these endosymbionts (Figs 3A and 4A). The reason could be their critical role in the RNAP complex formation and activity. Furthermore, all the endosymbionts preserve the catalytic core and its assembly domains within the β- and β’-subunits. Nevertheless, these present some changes in the domains involved in the binding with the σ and with other RNAP core subunits (S2 and S3 Figs).

The β flap-tip helix domain is incomplete in most *Hodgkinia* strains (Fig 3B, red box). This loss is evident in the comparisons of *Candidatus* Hodgkinia Dsem and TETUND2 with the 3-D crystal structure of the β-σ subunits complex in *E*. *coli* (Fig 3C–3E). Unlike *Candidatus* Hodgkinia Dsem, we can observe that TETUND2 does not present a complete β flap-tip helix domain (Fig 3D and 3E). These changes in the β flap-tip helix suggest that the RNAP core in these *Hodgkinia* cannot bind to the σ_4_ domain. Consequently, the σ factor should not bind to the -35-box promoter element properly. Mutants in *E*. *coli* lacking the β flap-tip helix result in an inability of the σ subunit to attach to the -35-box promoter without affecting the RNAP core to bind to DNA. Furthermore, these mutants adequately recognize the -10-box and the -10-extend promoter elements [19]. The absence of the β flap-tip helix domain, although not observed in bacteria, is common in archaea [70].

*Candidatus* Tremblaya phenacola PAVE and Carsonella ruddii CE conserve the β’ coiled-coil domain in the β’ subunit. In contrast, the rest of the endosymbionts display substitutions in at least one of the three necessary amino acids in the β’ coiled-coil (Fig 4B in brown, and S3 Fig). *In vitro* studies involving single amino acid substitutions in the β’ coiled-coil domain in the three residues ECO: R275Q, E295K, and A302D result in a deficient holoenzyme formation and a subsequent lack of promoter specificity [26]. Unlike Hodgkinia Dsem, the rest of Hodgkinias strains show the same substitutions in the two positions ECO: 275 and 302, and a deletion in the residue ECO 295. Furthermore, we observed that these substitutions could not affect the β’ coiled-coil domain formation in the re-created 3-D structures. However, the exposed residues and the orientation for the interaction with the σ_2_ domain are different from those in the *E*. *coli* β’ coiled-coil domain (Fig 4C–4E).

### The σ subunit shows the most erosive evolution in these endosymbionts

**Fig 5:**
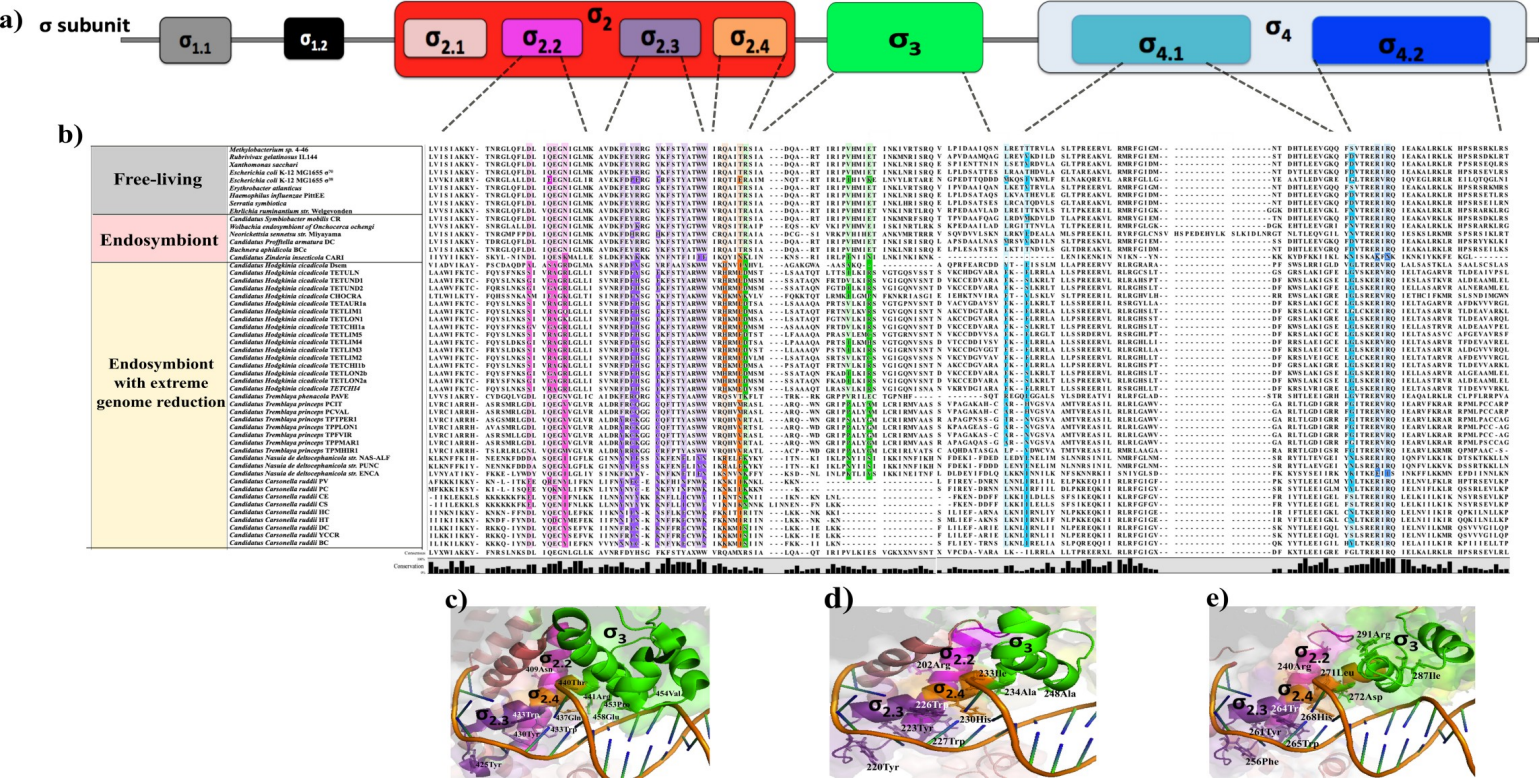
Domain conservation in the σ subunit and their variations in DNA-promoter recognition. **a)** The upper part shows the distribution of functional domains in the σ subunit: σ_2_ (red), σ_2.1_ (pink), σ_2.2_ (magenta), σ_2.3_ (violet), σ_2.4_ (orange), σ_3_ (green) and, σ_4_ (light blue), σ_4.1_ (cyan) and σ_4.2_ (dark blue). **b)** Amino acids alignment of σ subunits shows variations in the domains σ_2_ and σ_4_. Darker backgrounds show the functional residues that differ from those in the reference organism (*E*. *coli)*. **c)** Crystallographic structure of the *E*. *coli* σ subunit bound to DNA (4YLP). The σ_2.2_, σ_2.3_, σ_2.4_ subdomains and the σ_3_ domain are shown in magenta, violet, orange, and green, respectively. **d)** Predicted 3-D model of the σ subunit of Hodgkinia Dsem showing the subdomains and amino acids involved in recognizing and binding to the DNA**. e)** Predicted 3-D model of the σ subunit of Hodgkinia TETUND2 shows the subdomains and amino acids involved in recognizing and binding to DNA. The colors in **d)** and **e)** are the same that the homologs corresponding domains in **c)** for *E*. *coli*.

σ is the subunit that exhibits the most differentiated conservation among endosymbionts with extreme genome reduction (Fig 5A and 5B). In the case of *Candidatus* Tremblaya phenacola PAVE, it conserves whole the σ_2_ and σ_4_ domains. On the other side, *Candidatus* Hodgkinia, Nasuia deltocephalinicola, and Carsonella ruddii conserve the σ_4_ and, to a lesser extent, the σ_2_ domain (Fig 5B). The main variations happen inside the σ_2_ domain, whose amino acids interact with the -10-box promoter element and define the promoter specificity (Fig 5B, magenta, and orange amino acids).

For the σ_2.2_ subdomain, the Hodgkinia strains exhibit substitutions in all the four amino acids involved in the interaction with the β’ coiled-coil domain. The seventeen strains have the same substitutions for ECO E407A and 14 of them in ECO N409R. At the same time, each presents different amino acids at the positions ECO: 403 and ECO 406 (Fig 5B, magenta). Mutagenesis on these three amino acids in σ_2.2_ has shown that they cause just a weakening in their binding to the β’ coiled-coil domain [21]. Besides, thermal denaturation experiments indicate that these mutants folded differently concerning the *E*. *coli* wild type. Suggesting that these mutations’ principal effect is the allosteric regulation of the subdomains σ_2.3_ and σ_2.4_ who participate in DNA-melting and recognition of the -10-box promoter element [21]. On the other hand, all the endosymbionts have distinct variations in the σ_2.3_ subdomain. However, all of them conserve the essential aromatic residues necessary for DNA melting and the correct folding of the σ_2_ domain (Fig 5B, violet).

The σ_2.4_ subdomain presents substitutions on the amino acids involved in recognizing nucleotides at position -12 of the -10-box promoter element. Hodgkinia strains show a different amino acid at position ECO: 437. It consists of histidine instead of glutamine. Likewise, *Candidatus* Carsonella ruddii PV, PC, HT, HC, DC, YCCR, and BC have replaced the ECO: T440 with leucine or isoleucine. Tremblaya strains and *Candidatus* Tremblaya phenacola PAVE, conserve these two amino acids (ECO Q437 and T440) (Fig 5B, orange). Previous works studied punctual mutations in these regions of σ. More precisely, in the subdomains σ_2.4_ of *E*. *coli* σ^70^ and SigA from *Bacillus subtilis* (homologous to σ^70^). Changes in amino acids at these positions affect the specificity for their respective promoters [25]. In *E*. *coli*, substitutions in ECO: Q437H and T440I of σ^70^ result in conserving the capacity to recognize the nucleotide at the -12 position. However, promoters with cytosine in this position were significantly better (in specificity) than with another nucleotide [21]. Compared with *E*. *coli*, the substitutions observed in the subdomains of the σ_2_ domain seem not to affect the conformation of this domain. Nevertheless, the amino acids responsible for contacting the promoter can differ from those in *E*. *coli* (Fig 5C–5E).

The σ_3_ domain is well preserved in *Tremblaya phenacola* PAVE. However, it presents amino acid substitutions in other endosymbionts and is absent in *Carsonella ruddii*. In Hodgkinia, this domain is partially present in the Dsem strain, although more conserved in the rest (Fig 5D and 5E). The partial or total loss of the σ_3_ domain might suggest a deficient or null binding of σ at the -10-box extended promoter element.

### Most of the endosymbionts show strong purifying selection on the RNAP subunits core

We made two selection pressure analyses to investigate the effects of amino acid substitutions observed in genes encoding for the RNAP subunits. First, considering that bacterial endosymbionts are subject to an accelerated rate of molecular evolution [71]. We estimated the ratio of non-synonymous to synonymous substitutions (D_N_/D_S_) using phylogenetic codon-substitution models (S2 Table).

#### *Candidatus* Hodgkinia cicadicola

**Fig 6:**
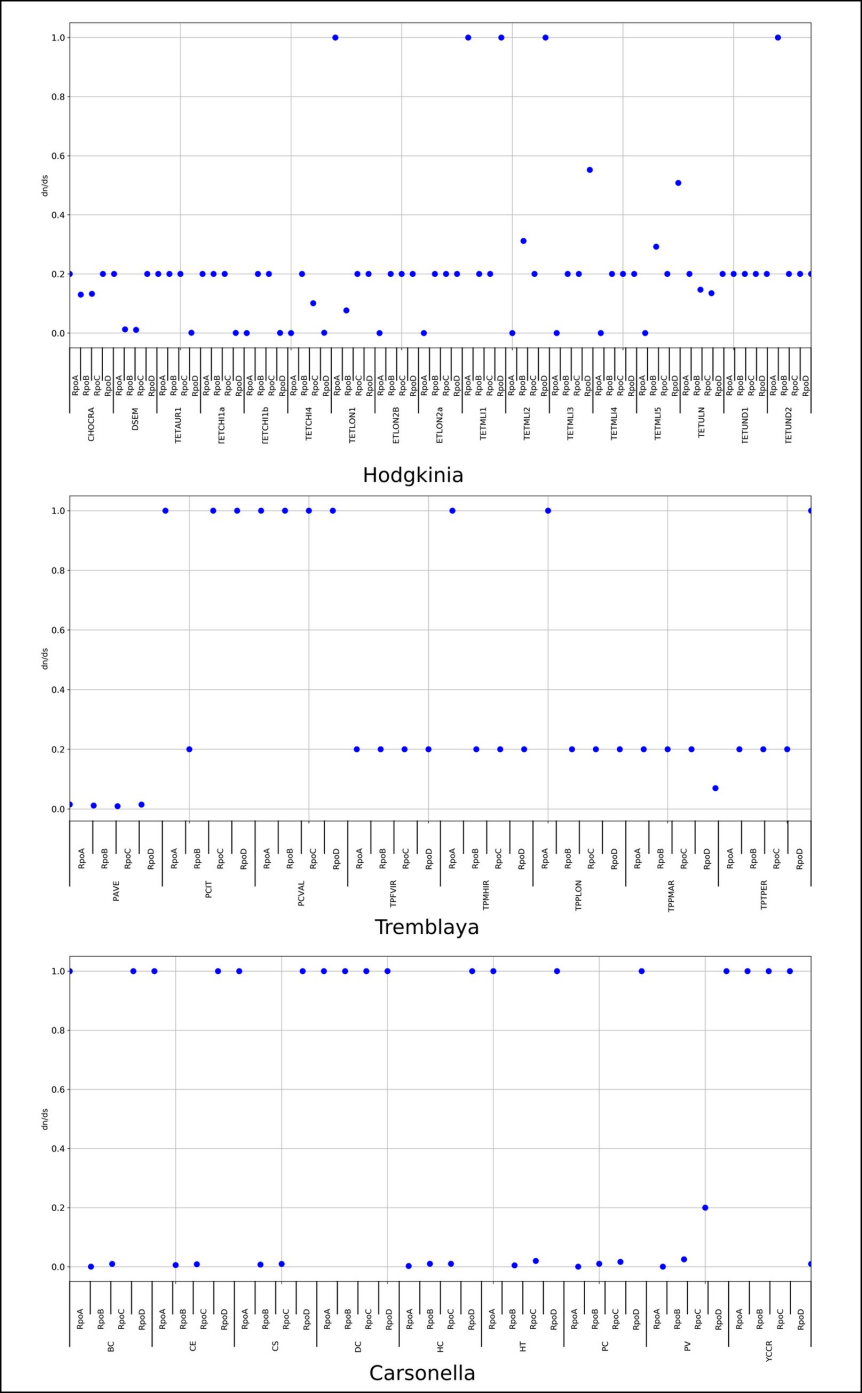
Selective pressure on the RNAP subunits by branch analysis. The figure shows the relationship D_N_/D_S_ for each subunit. Most genes are under negative selection, and only in few cases, they display values greater than 1 (represented as 1.2). However, this does not mean that they are under positive selection (level of significance greater than 0.05); in fact, the RNAP subunits show a relaxed selection in all the cases.

The *Candidatus* Hodgkinia cicadicola shows that their *rpoB* (β subunit) and *rpoC* (β**’**) were subject to purifying selection. With lower D_N_/D_S_ values (0.3), most of the nucleotide substitutions in these genes were synonymous. On the other hand, the *rpoA* (α subunit) genes had a higher purifying selection in most Hodgkinia strains (D_N_/D_S_<0.2). In contrast, Tetund 2, TETLON, and TETMLI1 strains present neutral selection (D_N_/D_S_ = 1). Finally, the *rpoD* gene (σ factor) shows an increased D_N_/D_S_ value (D_N_/D_S_<0.5). It means that the purifying selection is less rigorous in some Hodgkinia strains. This not uniform selection pressure could result in greater diversity in this subunit (Fig 6).

#### *Candidatus* Tremblaya

The strain PAVE, *rpoA*, *rpoB*, *rpoC*, and *rpoD* genes had ω values less than 0.02. Conversely, PCVAL shows a neutral selection for all the subunits (D_N_/D_S = 1_). So, a generalized relaxation of selective pressure, to a different extent, is present in these strains (Fig 6).

#### *Candidatus* Carsonella ruddii

The *rpoB* and *rpoC* genes in *Candidatus* Carsonella ruddii strains are more conserved than the other RNAP subunits. They had D_N_/D_S_ values less than 0.2 (except for DC and YCCR strains). The *rpoA* gene had a neutral selection in five strains (CE, CS, DC, HT, and YCCR) and a strong purifying selection in the rest of the *Candidatus* Carsonella strains (D_N_/D_S_<0.2). On the contrary, the *rpoD* gene had a neutral selection except for the BC strain (D_N_/D_S_ <0.01). This neutral selection might explain why the σ factor contains more amino acid variations concerning the other RNAP subunits (Fig 6).

### Positively selected amino acids are present in the α-NTD of the α subunit in the Hodgkinia strains

**Fig 7:**
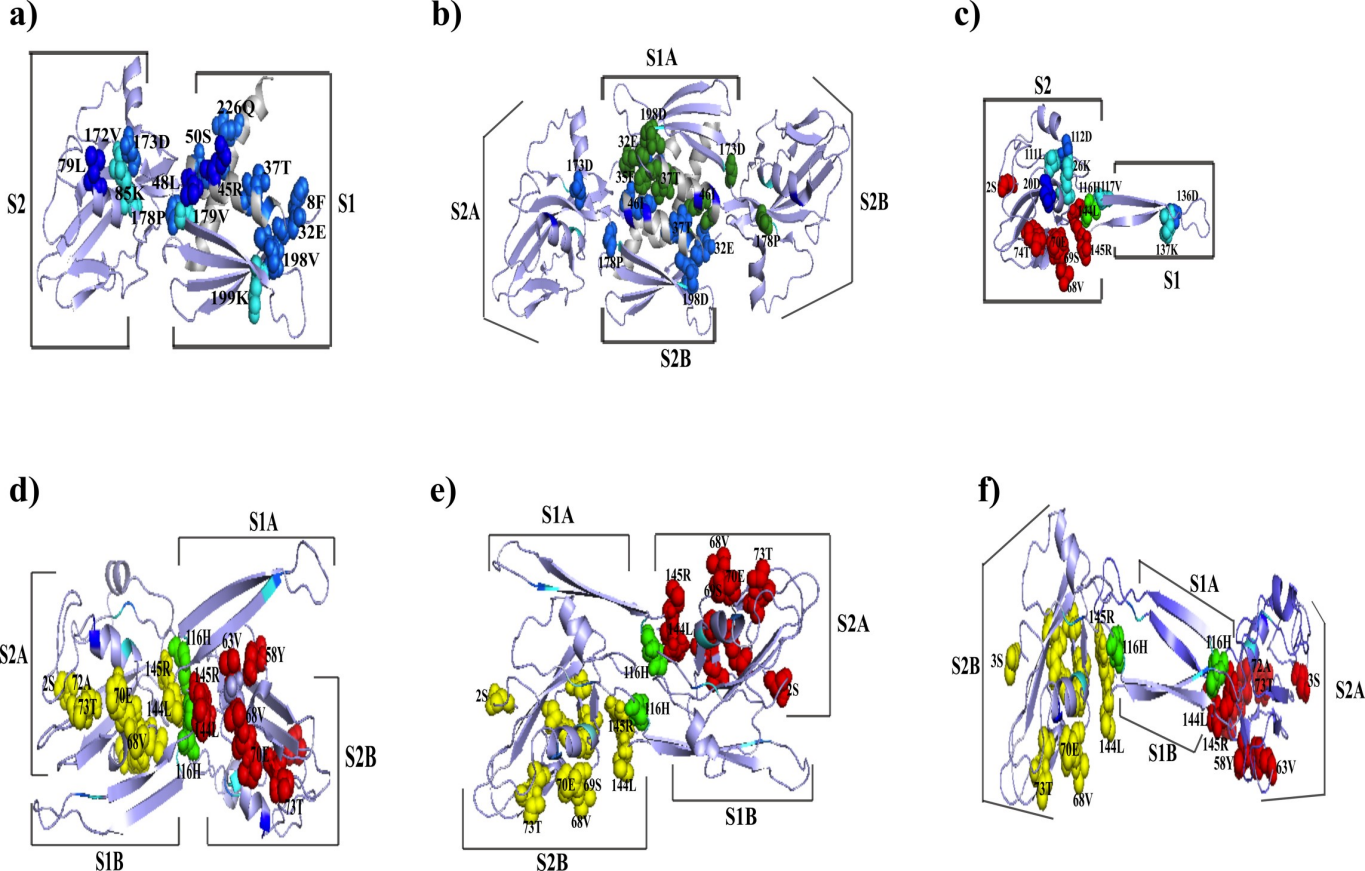
Structural comparison between the *E*. *coli* and the *Candidatus* Hodgkinia *TETUND2* α-subunits. **a**) 3-D structure of monomers and **b)** for homodimers of the α-NTD subunit in *E*. *coli* (4YLP). **c)** 3-D model of α-subunit monomer in *Candidatus* Hodgkinia TETUND2 with the amino acids under positive selection in red and the H116 in green. **d**), **e**), and **f**) show predictions of the α-subunit homodimer formation in *Candidatus* Hodgkinia TETUND2. The amino acids under positive selection are in red and yellow in each monomer, H116 in green. White regions in **a)** and **b)** structures are not conserved in the α-subunit of *Candidatus* Hodgkinia TETUND2. The amino acids in blue, dark, and turquoise are necessary for the RNAP core formation. In the **b)** structure, the dark green amino acids are the same as the blue in **a)**. S1 and S2 indicate subdomains 1 and 2 in the α-NTD. Moreover, the letter A and B correspond to each monomer that forms the homodimer.

Positively selected amino acids are present in the α-NTD of *Candidatus* Hodgkinia Dsem and TETUND2 (S3 Table). We mapped the selected amino acids with a high level of support (BEB p>0.95) in the structural models of the α subunit of *Candidatus* Hodgkinia TETUND2 and Dsem (Fig 7C, red amino acids, and S4 Fig, respectively). In *Candidatus* Hodgkinia Dsem, the selected amino acids V55, Q95, and H115 (S4 Fig) would be necessary for the correct folding of the α-NTD. In *Candidatus* Hodgkinia TETUND2, the amino acid residues V68, S69, and E70 allow adopting a similar structure to maintain the α subunit interactions with the β and β’ subunits in *E*. *coli* (Fig 7A and 7C).

As previously mentioned, the *Candidatus* Hodgkinia TETUND2 α subunit has lost part of the α-NTD involved in the homodimer formation. Therefore, we evaluated *in silico* if the *Candidatus* Hodgkinia TETUND2 α subunit can still form the homodimer, essential for RNAP core formation. We obtained three models with different clustered amino acids involved in homodimer formation (Fig 7D–7F). First, we mapped the sites under positive selection (Fig 7D–7F, amino acids red and yellow). Then, we performed *in silico* amino acid substitutions of the sites under positive selection. It was changing them by amino acids present in the same locations of different *Candidatus* Hodgkinia strains and *E*. *coli* (Fig 7B). We observed a substitution of histidine 116 by proline in the three models. This substitution changed the homodimer formation to be energetically unfavorable (S4 Table). Although H116 seems not under positive selection, it would have an essential role in stabilizing this strain’s homodimer formation.

### Variations on the conservation of the subunits involved in promoter recognition are independent of the CG content

Endosymbionts with reduced genomes carry out variable proportions of GC in their genomes. For example, while *Candidatus* Carsonella and *Candidatus* Nasuia contain less than 18% GC, *Candidatus* Hodgkinia and *Candidatus* Tremblaya contain above 40%. We want to know if such variations of GC content in genomes relate to changes in σ factors. Then, we carried out a comparative analysis that involved a phylogenetic tree (S5 Fig). We include the 37 strict endosymbiont bacteria of this study. However, we also include 13 homologs of σ^70^ coming from six endosymbionts and seven free-living bacteria. These other bacteria have a lesser extent of genome reduction. Still, similar, less, or more extensive GC contents than the endosymbionts studied (S5 Table).

Except for *C*. *Zinderia cicadicola*, the rest of the bacteria preserve the β flap-tip helix and the β’ coiled-coil domains. These also conserve the σ_2.2_ and σ_2.2_ subdomains involved in the promoter recognition in *E*. *coli* (Figs 3A, 4A and 5A). Thus, this analysis may suggest no relationship between the GC content and changes in the σ factors.

## Discussions

Here we approach the study of the evolution of RNAP subunits in vastly reduced bacteria genomes. We found that the β and β’ subunits are the most conserved in all the studied endosymbionts. These have just some differences in the regions involved in the interactions with the σ factor, possibly because of significant changes in σ. On the other side, the α subunit is more conserved in *Candidatus* Carsonella ruddii and Nasuia. In contrast, in the other endosymbionts, the α subunit has lost its α-CTD.

Furthermore, studies report the absence of a recognized gene encoding for the α subunit in Hodgkinia strains [37]. It is unknown how to perform an RNAP without the α subunit if the transcription is present in these Hodgkinias. It might mean that α subunits can follow ω as dispensable RNAP subunits. Hodgkinia strains inhabit their host as consortia with other bacteria. Then, it is attractive that the community consortia contribute to cellular activities [72]. Still, it isn’t easy to know if these complementary activities include gene transcription.

Furthermore, *Candidatus* Hodgkinia Dsem, CHOCRA, and TETULN strains preserve an α subunit and are not known to share their host with other Hodgkinia strains [37]. We also observed several amino acids under putative positive selection in the α subunit of *Candidatus* Hodgkinia TETUND2. Thus, they suggest compensation for the loss of critical amino acid regions for homodimer formation in this subunit.

Given the importance of σ in promoter recognition for transcription initiation, the substitutions observed in the σ_2.4_ subdomain might correspond to variations in the specificity of σ for promoters. Previous studies indicate that the substitution of some amino acids does not compromise their affinity to DNA. These include lysine, asparagine, serine, methionine, and phenylalanine [73]. However, these changes might exert mild effects on σ affecting its specificity for promoters. The differences observed in the σ_2.4_ subdomain of *Candidatus* Hodgkinia and *Candidatus* Nasuia correspond to mutations already experimentally found in the *E*. *coli* σ^70^ and *Bacillus* SigA [25,26,52].

So far, we can suggest the functionality of the RNAP of the seventeen Hodgkinia strains that conserve an α subunit, to the exception of the Dsem strain. These can recognize only the -10-box and the -10-box extended promoter elements. They lack the fragment required to form the β flap-tip helix domain that recognizes the -35-box promoter element and neither recognize the UP element (Figs 3D and 4D). *Candidatus* Tremblaya phenacola PAVE, Princeps PCIT, and PCVAL are the endosymbionts with a σ nearest to *E*. *coli* σ^38^ instead of the housekeeping σ^70^ (S4 Fig). σ^38^ pertains to the σ^70^ family, but in *E*. *coli*, it transcribes stationary phase genes [9]. The σ_4.1_ subdomain present in σ^38^ has some amino acid changes concerning those σ^70^. These changes make the β flap-tip helix domain of σ38 with more affinity and increased performance for the binding to -35-box promoter elements. This higher affinity to -35-box promoter elements and the stationary phase transcription factors could displace the main transcription activity from σ^70^ to σ^38^ in the stationary phase in *E*. *coli*.

In endosymbiotic bacteria with exceedingly reduced genomes, it has not been possible to locate σ^70^ canonical promoters. Not even for the most conserved, like the ribosomal genes [74]. This inability to find promoters might be partly due to the high A+T percentage and the lack of intergenic regions in these genomes. However, this may not be the whole explanation since Hodgkinia and Tremblaya have relatively high G+C %. More than 90% of their genome comprises coding sequences, and neither presents a recognized promoter [7]. Hence, these results suggest that RNAP in endosymbionts can conserve some sequence recognition capacity, but this should differ from the σ^70^ consensus promoters in *E*. *coli*. Thus, it seems that some promoter elements are unnecessary in endosymbiont. With this, shorter promoter sequences might be sufficient for gene transcription. This fact can explain the difficulty of recovering consensus promoter sequences as we know in free-living bacteria. Besides, transcription factors that assist in gene regulation are also absent in these bacteria with highly reduced genomes, being the last to be lost the nucleoid-associated proteins [36]. Therefore, it makes sense that regions for gene activation, such as the UP-promoter element and the contact region for these activators in the α subunit, are absent. Variations in recognition regions of promoters might not be the only ones in these bacteria. For example, previous reports indicate significant changes in the 16S ribosomal 3’ tail and its binding sequence with the corresponding changes in the Shine-Dalgarno element localized upstream of the protein-encoding genes [75]. Then, the observations of this study can be a more generalized phenomenon in these bacteria.

## Conclusions

DNA sequences encoding for each of the RNAP subunits exhibit a reduction of 16% on average compared to those in *E*. *coli*. The gene reductions present in RNAP subunits are independent of the CG content (18–40% GC in these genomes). Most endosymbionts experiment strong purifying selection on the RNAP subunit genes, particularly on the β and β’ subunits. In the case of σ, the type of selection determined was less uniform among the endosymbionts.

A closer inspection in the α subunit reveals that the α-NTD is more conserved than α-CTD. Additionally, some amino acid changes in homodimer assembly are under positive selection in the *Candidatus* Hodgkinia TETUND2 and Dsem strains. On the other hand, the β and β’ subunits are more conserved in strict endosymbionts except for the β flap-tip helix domain in Hodgkinia strains. Furthermore, the σ subunit presents the more variated erosion in these endosymbionts. These unequal losses result in promoter elements’ differential recognition.

**Fig 8:**
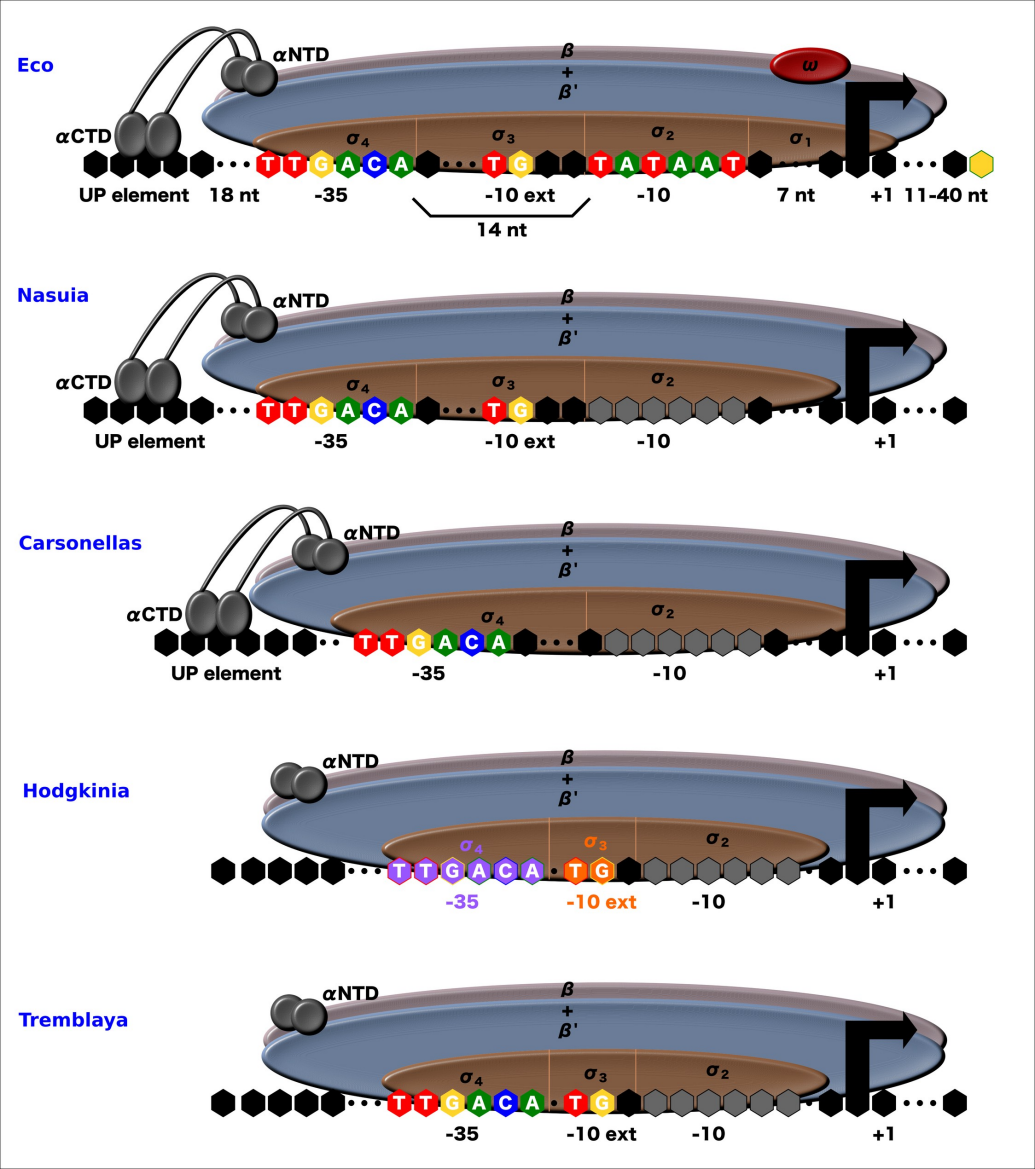
Proposed functional RNAP models of bacterial endosymbionts with reduced genomes. The conserved domains in each group of bacteria are present in each illustration. Figures correspond to *E*. *coli* RNAP **(**Eco**),** *Nasuia* strains RNAP, *and Carsonella* strains RNAP. In Hodgkinia strains in orange, the TETULN, TETUND1, and TETUND1 strains use the -10 extended region. In purple, Dsem strains recognize the -35 element. *Candidatus* Tremblaya phenacola PAVE, Princeps PCIT, and PCVAL RNAP model (Based on [10, 76]).

To better illustrate our inferences, we present a functional conclusion based on the conservation of RNAP subunits. We offer drawing models with the inferred regions of promoters where RNAP for each endosymbiont should be recognizing (Fig 8). We can deduce that the RNAP of *Nasuia* conserved the more significant similarity to the *E*. *coli* σ^70^. According to this, *Nasuia* RNAP should recognize the two main promoter elements (-10-box and -35-box) and the two promoter accessory elements (-10-box extended and even the UP element). Another way, Carsonella RNAP seems to maintain recognition of the promoter elements except for the -10-box extended element. This limited recognition can result in promoters with shorter regions between the -10-box and the -35-box promoter elements. In another case, the σ of Tremblaya resembles more to σ^38^ instead of the canonical σ^70^. In Tremblaya and Hodgkinia, due to the absence of the α-CTD, they might not recognize the UP element. And in the case of the strain *Candidatus* Hodgkinia Dsem neither the -10 extended promoter element. Additional studies, ideally experimental ones, should generate new knowledge about what is happening with the functioning of shorter proteins in this fascinating field of highly reduced genomes.

## Supporting information

S1 Fig Interaction domains in α subunit alignment. (TIF)

S2 Fig Interaction domains in the β subunit alignment. (TIF)

S3 Fig Functional domains and amino acids in β’ subunit alignment. (TIF)

S4 Fig Selected sites mapped in *Hodgkinia* Dsem α subunit. (TIF)

S5 Fig Phylogenetic tree of sigma 70 proteins in endosymbionts with extreme genome reduction and other symbionts and free-living bacteria. (TIF)

S1 Table Complete list of studied endosymbionts. (PDF)

S2 Table Bacteria with similar %GC to endosymbiotic bacteria with reduced genomes. (PDF)

S3 Table Results obtained by selective pressure analysis by branch model. (PDF)

S4 Table Results of selective pressure obtained by the branch-site model. (PDF)

S5 Table Changes of free energy by in silico mutations in selected sites of α subunit homodimer predicted for Hodgkinia TETUND2. (PDF)
