# Supplementary figures and images for: RNA polymerases in strict endosymbiont bacteria with extreme genome reduction show distinct erosions that might result in limited and differential promoter recognition
Source: PLoS One. 2021 Jul 29;16(7):e0239350. doi: 10.1371/journal.pone.0239350 (PMC8321222; doi:10.1371/journal.pone.0239350)

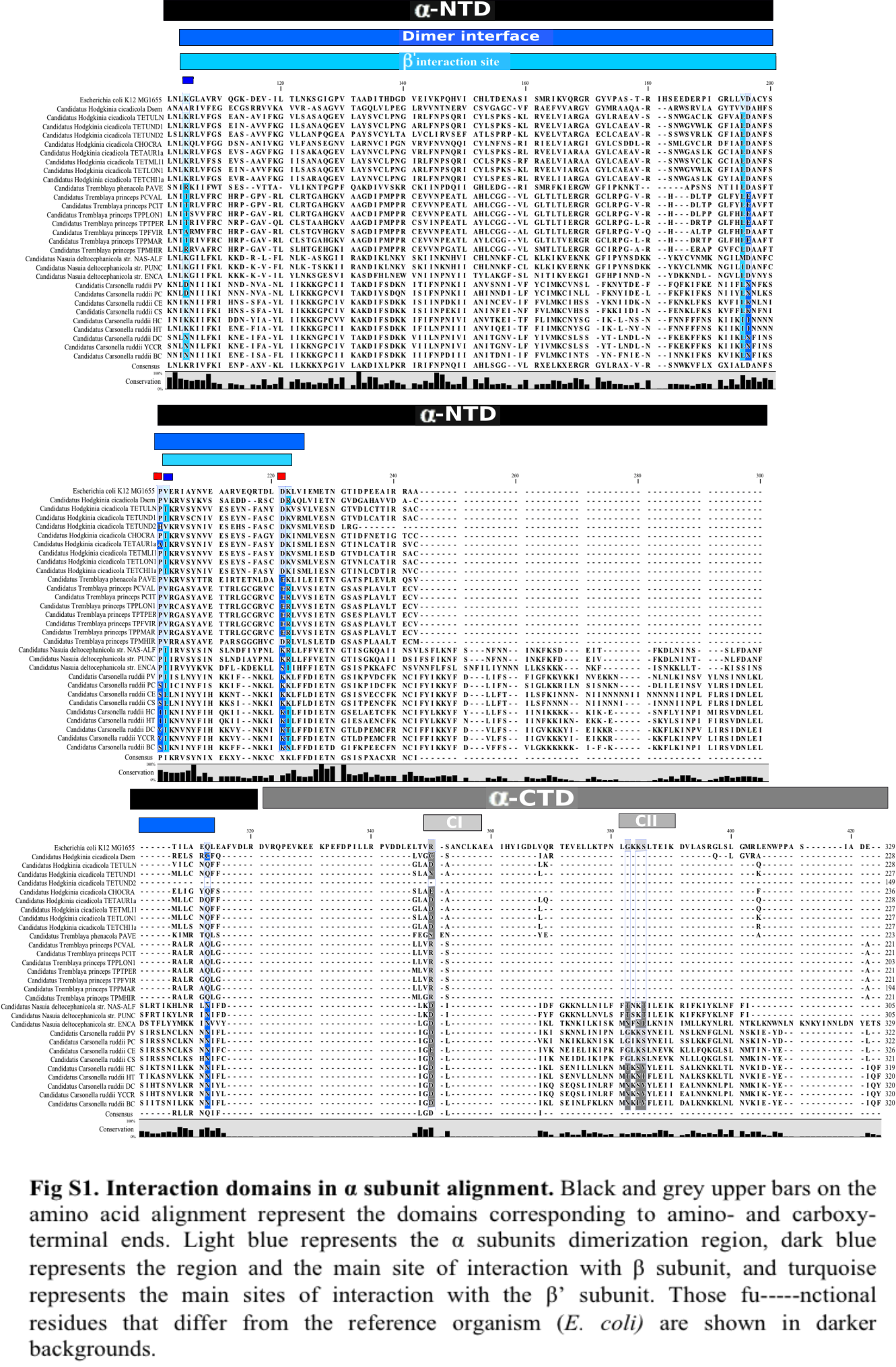

Supplement: S1 Fig — (TIF) [file pone.0239350.s001.tif]

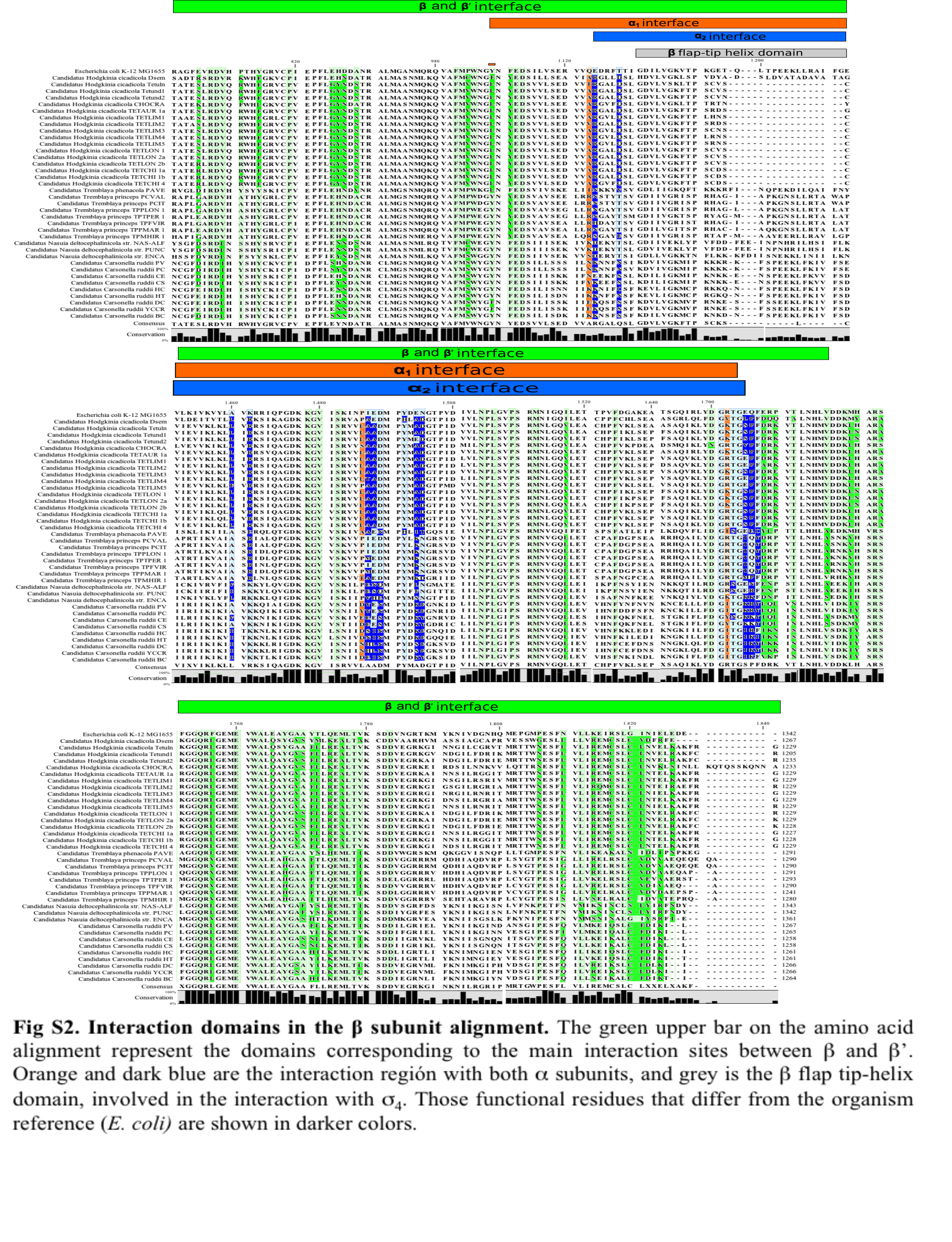

Supplement: S2 Fig — (TIF) [file pone.0239350.s002.tif]

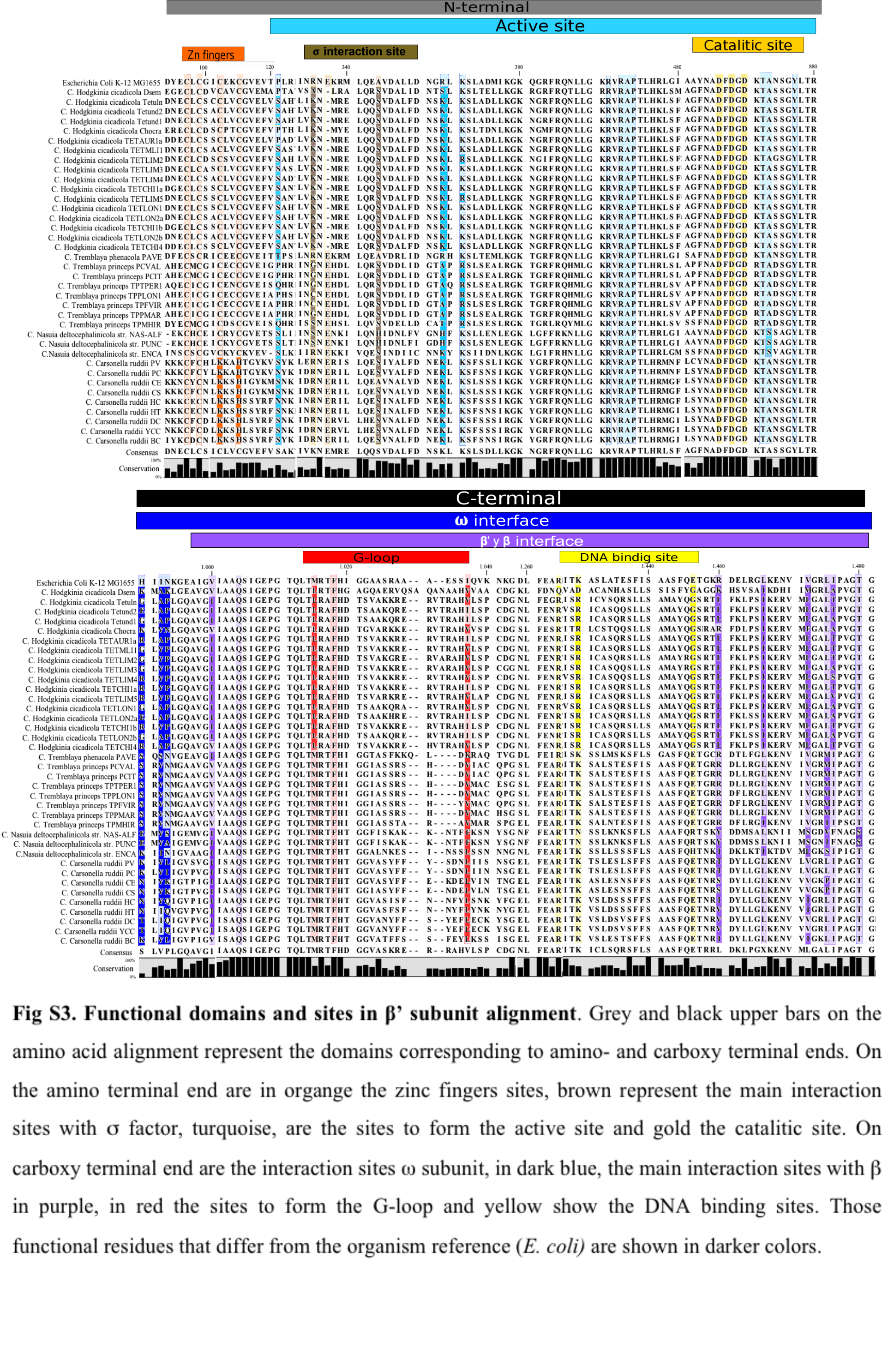

Supplement: S3 Fig — (TIF) [file pone.0239350.s003.tif]

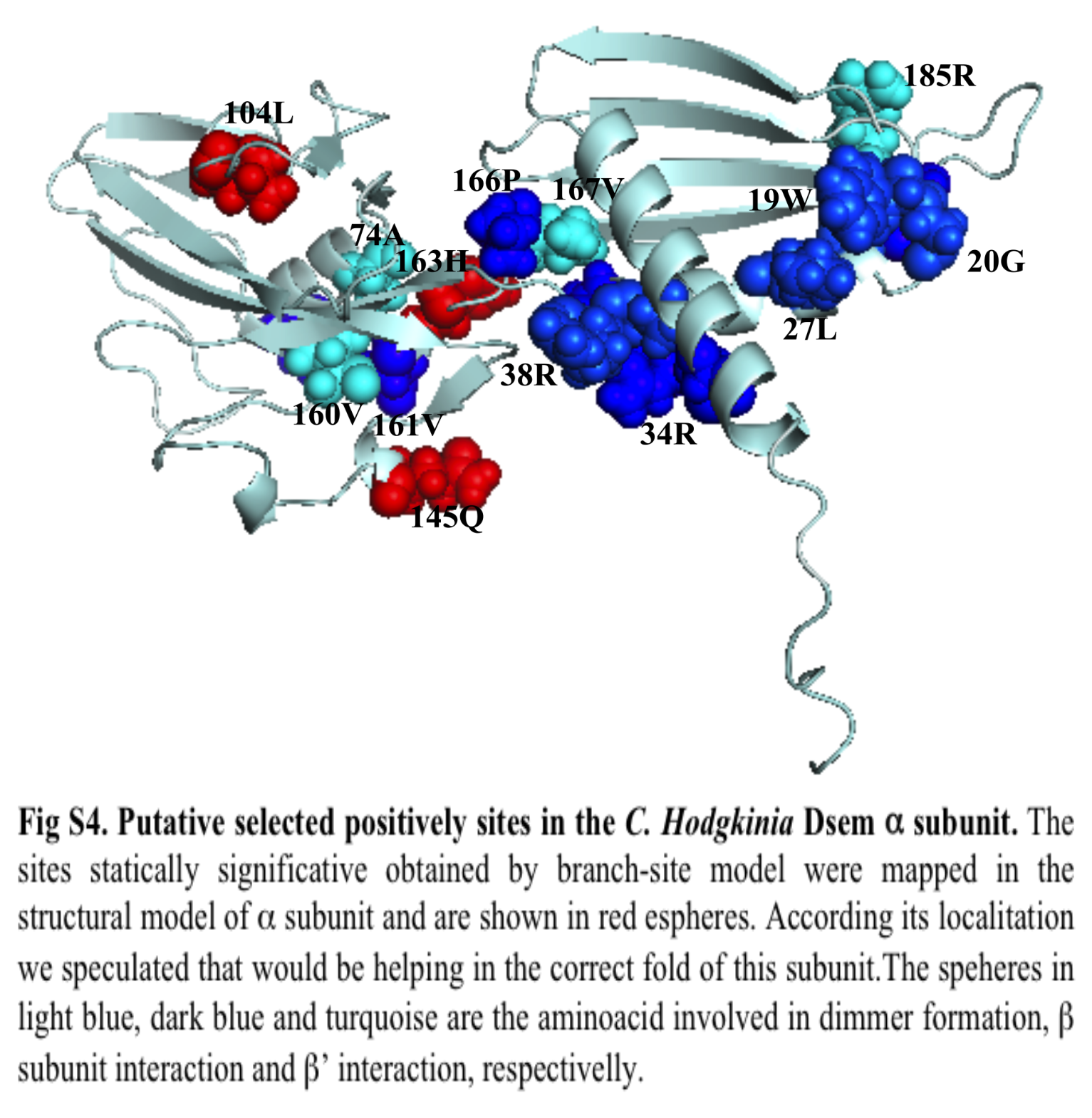

Supplement: S4 Fig — (TIF) [file pone.0239350.s004.tif]

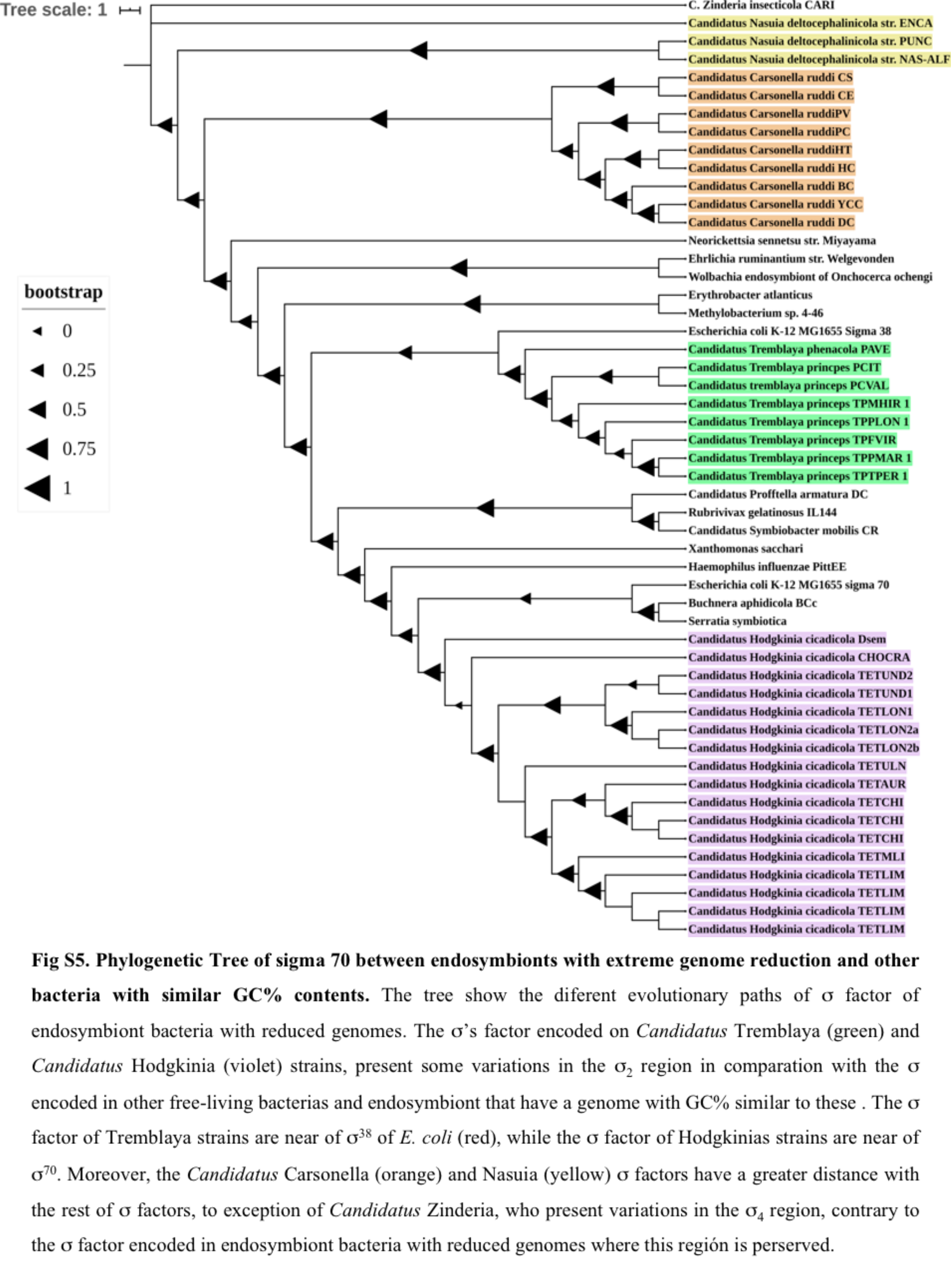

Supplement: S5 Fig — (TIF) [file pone.0239350.s005.tif]
