# Supplementary material for: RNA polymerases in strict endosymbiont bacteria with extreme genome reduction show distinct erosions that might result in limited and differential promoter recognition
Source: PLoS One. 2021 Jul 29;16(7):e0239350. doi: 10.1371/journal.pone.0239350 (PMC8321222; doi:10.1371/journal.pone.0239350)
Supplement: S1 Table — (PDF) [file pone.0239350.s006.pdf]

Table S1. Complete list of studied endosymbionts

Name	Habitat	Genome size[a]	Genes encoded[b]	%GC[c]	DOI[d]
Escherichia coli k-12 MG1655	Free-living	4.638	4405	50.8	10.1126/science.277.5331.1453
**Alpha proteobacteria**					
Candidatus Hodgkinia cicadicola Tetund1	Endosymbiont	0.133	121	46.8	10.1016/j.cell.2014.07.047
Candidatus Hodgkinia cicadicola Tetund2	Endosymbiont	0.140	140	46.2	10.1016/j.cell.2014.07.047
Candidatus Hodgkinia cicadicola Tetuln	Endosymbiont	0.140	140	45.4	10.1016/j.cell.2014.07.047
Candidatus Hodgkinia cicadicola Dsem	Endosymbiont	0.143	169	58.4	10.1073/pnas.0906424106
Candidatus Hodgkinia cicadicola TETLIM1	Endosymbiont	0.145	184	45.4	10.1073/pnas.1712321115
Candidatus Hodgkinia cicadicola TETLIM2	Endosymbiont	0.130	159	45.1	10.1073/pnas.1712321115
Candidatus Hodgkinia cicadicola TETLIM3	Endosymbiont	0.128	138	47.8	10.1073/pnas.1712321115
Candidatus Hodgkinia cicadicola TETLIM4	Endosymbiont	0.126	147	47.2	10.1073/pnas.1712321115
Candidatus Hodgkinia cicadicola TETLIM5	Endosymbiont	0.121	145	45.8	10.1073/pnas.1712321115

Candidatus Hodgkinia cicadicola CHOCRA	Endosymbiont	0.148	194	38.7	10.1073/pnas.1712321115
Candidatus Hodgkinia cicadicola TETCHI 1a	Endosymbiont	0.129	163	44.9	10.1073/pnas.1712321115
Candidatus Hodgkinia cicadicola TETCHI 1b	Endosymbiont	0.129	156	44.8	10.1073/pnas.1712321115
Candidatus Hodgkinia cicadicola TETCHI 2a	Endosymbiont	0.116	115	45.8	10.1073/pnas.1712321115
Candidatus Hodgkinia cicadicola TETCHI 4	Endosymbiont	0.105	114	45.6	10.1073/pnas.1712321115
Candidatus Hodgkinia cicadicola TETLON 1	Endosymbiont	0.133	158	47.7	10.1073/pnas.1712321115
Candidatus Hodgkinia cicadicola TETLON 2a	Endosymbiont	0.140	179	46.5	10.1073/pnas.1712321115
Candidatus Hodgkinia cicadicola TETLON 2b	Endosymbiont	0.137	176	46.7	10.1073/pnas.1712321115
Candidatus Hodgkinia cicadicola TETAUR 1a	Endosymbiont	0.125	157	46.3	10.1073/pnas.1712321115
Beta proteobacteria					
Candidatus Tremblaya phenacola PAVE	Endosymbiont	0.171	183	42.1	10.1016/j.cell.2013.05.040

Candidatus Tremblaya princeps PCIT	Endosymbiont	0.138	172	58.8	10.1016/j.cub.2011.06.051
Candidatus Tremblaya princeps PCVAL	Endosymbiont	0.138	174	58.8	10.1128/JB.05749-11
Candidatus Tremblaya princeps TPPLON 1	Endosymbiont	0.144	179	58.9	10.1073/pnas.1603910113
Candidatus Tremblaya princeps TPTPER 1	Endosymbiont	0.143	174	57.8	10.1073/pnas.1603910113
Candidatus Tremblaya princeps TPFVIR	Endosymbiont	0.141	173	58.2	10.1073/pnas.1603910113
Candidatus Tremblaya princeps TPPMAR 1	Endosymbiont	0.140	170	58.3	10.1073/pnas.1603910113
Candidatus Tremblaya princeps TPMHIR 1	Endosymbiont	0.138	168	61.8	10.1073/pnas.1603910113
Candidatus Nasuia deltocephalinicola str. NAS-ALF	Endosymbiont	0.112	137	17.1	10.1093/gbe/evt118
Candidatus Nasuia deltocephalinicola str. PUNC	Endosymbiont	0.112	137	16.6	10.1128/genomeA.01604-15
Candidatus Nasuia deltocephalinicola str. ENCA	Endosymbiont	0.144	192	15.2	10.1093/gbe/evx134
Gamma proteobacteria					
Candidatus Carsonella ruddii HT isolate Thao2000	Endosymbiont	0.157	166	14.6	10.1093/molbev/mss180
Candidatus Carsonella ruddii PV	Endosymbiont	0.159	175	16.6	10.1126/science.1134196
Candidatus	Endosymbiont	0.159	174	15.6	10.1093/molbev/mss180

Carsonella ruddii PC isolate NHV					
Candidatus Carsonella ruddii CS isolate Thao2000	Endosymbiont	0.162	181	14.0	10.1093/molbev/mss180
Candidatus Carsonella ruddii CE isolate Thao2000	Endosymbiont	0.162	182	14.0	10.1093/molbev/mss180
Candidatus Carsonella ruddii HC isolate Thao2000	Endosymbiont	0.166	182	14.2	10.1093/molbev/mss180
Candidatus Carsonella ruddii DC	Endosymbiont	0.174	196	17.6	10.1016/j.cub.2013.06.027
Candidatus Carsonella ruddii YCCR	Endosymbiont	0.174	224	17.6	10.1128/genomeA.01316-15
Candidatus Carsonella ruddii BC	Endosymbiont	0.173	227	14.8	10.1128/genomeA.01466-17

[a]Genome size in Megabase pairs, [b]Open reading frame, [c]The GC percent content in the genome [d]Digital object identifier to access the article of reference on the web.
