# Supplementary material for: RNA polymerases in strict endosymbiont bacteria with extreme genome reduction show distinct erosions that might result in limited and differential promoter recognition
Source: PLoS One. 2021 Jul 29;16(7):e0239350. doi: 10.1371/journal.pone.0239350 (PMC8321222; doi:10.1371/journal.pone.0239350)
Supplement: S2 Table — (PDF) [file pone.0239350.s007.pdf]

Table S2. Results obtained of selective pressure analysis by branch model

Test branch model[a]	np[b]	lnL[c]	vs[d]	lnLc[e]	2ΔlnLc[f]	npt[g]	Dist.Chi2[h]	ω estimated[i]
One Model Hodgkinia RpoA	17	-2471.287166	Free model	-18.507884	37.015768	14	1.17178E-09	
Free Model Hodgkinia RpoA	31	-2446.872437						
Hodgkinia CHOCRA RpoA fix 0.2	17	-2470.31546	Two ratio model	-0.008425	0.01685	1	0.89671869	
Hodgkinia CHOCRA RpoA fix 1	17	-2489.79505	Two ratio model	19.488015	38.97603	1	4.29041E-10	
Hodgkinia CHOCRA RpoA fix 1.2	17	-2494.262381	Two ratio model	-23.955346	47.910692	1	4.46082E-12	
Two ratio model Hodgkinia CHOCRA rpoA	18	-2470.307035	One model	0.980131	1.960262	1	0.161485301	0.19283
Hodgkinia Dsem RpoA fix 0.2	17	-2470.236654	Two ratio model	-1.361362	2.722724	1	0.098929054	
Hodgkinia Dsem RpoA fix 1	17	-2481.725511	Two ratio model	-12.850219	25.700438	1	3.98737E-07	
Hodgkinia Dsem RpoA fix 1.2	17	-2484.432642	Two ratio model	-15.55735	31.1147	1	2.43222E-08	
Two ratio model Hodgkinia Dsem rpoA	18	-2468.875292	One model	2.411874	4.823748	1	0.028070245	0.07483
Hodgkinia TETAUR1a RpoA fix 0.2	17	-2471.424532	Two ratio model	-0.142973	0.285946	1	0.592830219	
Hodgkinia TETAUR1a RpoA fix 1	17	-2472.616728	Two ratio model	-1.335169	2.670338	1	0.10223431	
Hodgkinia TETAUR1a RpoA fix 1.2	17	-2472.977233	Two ratio model	-1.695674	3.391348	1	0.065539347	
Two ratio model Hodgkinia TETAUR1a rpoA	18	-2471.281559	One model	0.005607	0.011214	1	0.915664728	0.29228
Hodgkinia TETCHi1a RpoA fix 0.2	17	-2470.270921	Two ratio model	-0.647361	1.294722	1	0.255179566	
Hodgkinia TETCHI1aRpoA fix 1	17	-2479.610465	Two ratio model	-9.986905	19.97381	1	7.85102E-06	
Hodgkinia TETCHI1aRpoA fix 1.2	17	-2481.251735	Two ratio model	-11.628175	23.25635	1	1.41782E-06	
Two ratio model Hodgkinia TETCHI1a rpoA	18	-2469.62356	One model	1.663606	3.327212	1	0.068142293	0.11162
Hodgkinia TETLON1RpoA fix 0.2	17	-2471.139759	Two ratio model	-0.11582	0.23164	1	0.630310451	
Hodgkinia TETLON1RpoA fix 1	17	-2472.289941	Two ratio model	-1.266002	2.532004	1	0.111558454	
Hodgkinia TETLON1RpoA fix 1.2	17	-2472.415813	Two ratio model	-1.391874	2.783748	1	0.095225086	
Two ratio model Hodgkinia TETLON1 RpoA	18	-2471.023939	One model	0.263227	0.526454	1	0.468101499	0.10491
Hodgkinia TETMLI1RpoA fix 0.2	17	-2471.021625	Two ratio model	-0.249707	0.499414	1	0.479757719	
Hodgkinia TETMLI1RpoA fix 1	17	-2471.780427	Two ratio model	-1.008509	2.017018	1	0.155544345	

Hodgkinia TETMLI1RpoA fix 1.2	17	-2471.791634	Two ratio model	-1.019716	2.039432	1	0.153266867	
Two ratio model Hodgkinia TETMLI1 RpoA	18	-2470.771918	One model	0.515248	1.030496	1	0.3100422	0.09951
Hodgkinia Tetuln RpoA fix 0.2	17	-2470.405699	Two ratio model	-0.185239	0.370478	1	0.542743832	
Hodgkinia TetulnRpoA fix 1	17	-2480.944495	Two ratio model	-10.724035	21.44807	1	3.63542E-06	
Hodgkinia TetulnRpoA fix 1.2	17	-2482.917828	Two ratio model	-12.697368	25.394736	1	4.67191E-07	
Two ratio model Hodgkinia Tetuln RpoA	18	-2470.22046	One model	1.066706	2.133412	1	0.14411964	0.1569
Hodgkinia Tetund1RpoA fix 0.2	17	-2470.773845	Two ratio model	-0.267688	0.535376	1	0.464355405	
Hodgkinia Tetund1RpoA fix 1	17	-2475.935776	Two ratio model	-5.429619	10.859238	1	0.000983041	
Hodgkinia Tetund1RpoA fix 1.2	17	-2476.794288	Two ratio model	-6.288131	12.576262	1	0.000390677	
Two ratio model Hodgkinia Tetund1 RpoA	18	-2470.506157	One model	0.781009	1.562018	1	0.211369991	0.12383
Hodgkinia Tetund2RpoA fix 0.2	17	-2474.156893	Two ratio model	-23.25098	46.50196	1	9.15269E-12	
Hodgkinia Tetund2RpoA fix 1	17	-2451.42513	Two ratio model	0.519217	1.038434	1	0.308185962	
Hodgkinia Tetund2RpoA fix 1.2	17	-2450.989321	Two ratio model	0.083408	0.166816	1	0.682957172	
Two ratio model Hodgkinia Tetund2 RpoA	18	-2450.905913	One model	-20.381253	40.762506	1	1.719E-10	1.35978
One Model Hodgkinia RpoB	33	-23670.58811	Free model	74.618733	149.237466	30	9.15398E-18	
Free Model Hodgkinia RpoB	63	-23595.96938						
Hodgkinia Chocra RpoB fix 0.2	33	-23671.16575	Two ratio model	-2.509783	5.019566	1	0.02506245	
Hodgkinia Chocra RpoB fix 1	33	-23695.998	Two ratio model	-27.342036	54.684072	1	1.41549E-13	
Hodgkinia Chocra RpoB fix 1.2	33	-23697.70329	Two ratio model	-29.047326	58.094652	1	2.49804E-14	
Two ratio model Hodgkinia Chocra RpoD	34	-23668.65597	One model	1.932144	3.864288	1	0.049324117	0.13002
Hodgkinia Dsem RpoB fix 0.2	33	-23671.848	Two ratio model	-38.867049	77.734098	1	1.17889E-18	
Hodgkinia Dsem RpoB fix 1	33	-23779.62205	Two ratio model	-146.641098	293.282196	1	9.58096E-66	
Hodgkinia Dsem RpoB fix 1.2	33	-23795.32928	Two ratio model	-162.348329	324.696658	1	1.3737E-72	
Two ratio model Hodgkinia Dsem RpoD	34	-23632.98095	One model	37.607158	75.214316	1	4.22294E-18	0.01247
Hodgkinia TETAUR1a RpoB fix 0.2	33	-23670.29986	Two ratio model	-0.183601	0.367202	1	0.544533382	
Hodgkinia TETAUR1a RpoB fix 1	33	-23704.76845	Two ratio model	-34.652198	69.304396	1	8.43813E-17	
Hodgkinia TETAUR1a RpoB fix 1.2	33	-23712.80791	Two ratio model	-42.691652	85.383304	1	2.45787E-20	

Two ratio model Hodgkinia TETAUR1a RpoD	34	-23670.11625	One model	0.471858	0.943716	1	0.33132401	0.22293
Hodgkinia TETCHI1a RpoB fix 0.2	33	-23670.72087	Two ratio model	-0.721921	1.443842	1	0.229518632	
Hodgkinia TETCHI1a RpoB fix 1	33	-23679.78743	Two ratio model	-9.788476	19.576952	1	9.66279E-06	
Hodgkinia TETCHI1a RpoB fix 1.2	33	-23681.32933	Two ratio model	-11.330382	22.660764	1	1.93274E-06	
Two ratio model Hodgkinia TETCHI1a RpoD	34	-23669.99895	One model	0.589161	1.178322	1	0.277697868	0.10374
Hodgkinia TETCHI1b RpoB fix 0.2	33	-23670.61711	Two ratio model	-0.042853	0.085706	1	0.769708761	
Hodgkinia TETCHI1b RpoB fix 1	33	-23677.2213	Two ratio model	-6.647046	13.294092	1	0.000266244	
Hodgkinia TETCHI1b RpoB fix 1.2	33	-23678.54095	Two ratio model	-7.966692	15.933384	1	6.56112E-05	
Two ratio model Hodgkinia TETCHI1b RpoB	34	-23670.57426	One model	0.013857	0.027714	1	0.86778298	0.17331
Hodgkinia TETCHI4 RpoB fix 0.2	33	-23669.97991	Two ratio model	-1.261746	2.523492	1	0.11216196	
Hodgkinia TETCHI4 RpoB fix 1	33	-23675.90202	Two ratio model	-7.183848	14.367696	1	0.00015036	
Two ratio model Hodgkinia TETCHI4 RpoB	34	-23668.71817	One model	1.869945	3.73989	1	0.053127943	0.27786
Hodgkinia TETLIM2 RpoB fix 0.2	33	-23669.26692	Two ratio model	-3.598084	7.196168	1	0.007305942	
Hodgkinia TETLIM2 RpoB fix 1	33	-23688.82814	Two ratio model	-23.159308	46.318616	1	1.00505E-11	
Hodgkinia TETLIM2 RpoB fix 1.2	33	-23695.92062	Two ratio model	-30.251786	60.503572	1	7.34451E-15	
Two ratio model Hodgkinia TETLIM2 RpoB	34	-23665.66884	One model	4.919276	9.838552	1	0.00170892	0.31207
Hodgkinia TETLIM3 RpoB fix 0.2	33	-23670.85554	Two ratio model	-0.444421	0.888842	1	0.345791308	
Hodgkinia TETLIM3 RpoB fix 1	33	-23725.91068	Two ratio model	-55.49956	110.99912	1	5.9197E-26	
Hodgkinia TETLIM3 RpoB fix 1.2	33	-23737.00375	Two ratio model	-66.592626	133.185252	1	8.23569E-31	
Two ratio model Hodgkinia TETLIM3 RpoB	34	-23670.41112	One model	0.17699	0.35398	1	0.551868749	0.17027
Hodgkinia TETLIM4 RpoB fix 0.2	33	-23670.05933	Two ratio model	-0.706356	1.412712	1	0.234606707	
Hodgkinia TETLIM4 RpoB fix 1	33	-23699.97876	Two ratio model	-30.625779	61.251558	1	5.02288E-15	
Hodgkinia TETLIM4 RpoB fix 1.2	33	-23707.70899	Two ratio model	-38.356009	76.712018	1	1.97796E-18	
Two ratio model Hodgkinia TETLIM4 RpoB	34	-23669.35298	One model	1.235135	2.47027	1	0.116017994	0.24684
Hodgkinia TETLIM5 RpoB fix 0.2	33	-23669.56412	Two ratio model	-2.445462	4.890924	1	0.026998233	
Hodgkinia TETLIM5 RpoB fix 1	33	-23691.00667	Two ratio model	-23.888008	47.776016	1	4.77802E-12	
Hodgkinia TETLIM5 RpoB fix 1.2	33	-23697.83719	Two ratio model	-30.718532	61.437064	1	4.57124E-15	

Two ratio model Hodgkinia TETLIM5 RpoB	34	-23667.11866	One model	3.469455	6.93891	1	0.008434049	0.29257
Hodgkinia TETLON1 RpoB fix 0.2	33	-23671.20349	Two ratio model	-5.277765	10.55553	1	0.00115841	
Hodgkinia TETLON1 RpoB fix 1	33	-23709.6153	Two ratio model	-43.689579	87.379158	1	8.95896E-21	
Hodgkinia TETLON1 RpoB fix 1.2	33	-23715.84192	Two ratio model	-49.916201	99.832402	1	1.65855E-23	
Two ratio model Hodgkinia TETLON1 RpoB	34	-23665.92572	One model	4.662389	9.324778	1	0.002260757	0.07659
Hodgkinia TETLON2a RpoB fix 0.2	33	-23670.66333	Two ratio model	-0.171124	0.342248	1	0.558534499	
Hodgkinia TETLON2a RpoB fix 1	33	-23680.81244	Two ratio model	-10.32023	20.64046	1	5.54126E-06	
Hodgkinia TETLON2a RpoB fix 1.2	33	-23682.79044	Two ratio model	-12.298236	24.596472	1	7.0681E-07	
Two ratio model Hodgkinia TETLON2a RpoB	34	-23670.49221	One model	0.095905	0.19181	1	0.661414421	0.15653
Hodgkinia TETLON2b RpoB fix 0.2	33	-23670.41903	Two ratio model	-0.550495	1.10099	1	0.294048943	
Hodgkinia TETLON2b RpoB fix 1	33	-23673.54912	Two ratio model	-3.680585	7.36117	1	0.006664741	
Hodgkinia TETLON2b RpoB fix 1.2	33	-23674.67241	Two ratio model	-4.803879	9.607758	1	0.001937571	
Two ratio model Hodgkinia TETLON2b RpoB	34	-23669.86853	One model	0.719579	1.439158	1	0.230275643	0.3106
Hodgkinia TETMLI1 RpoB fix 0.2	33	-23669.94498	Two ratio model	1.418017	2.836034	1	0.092171455	
Hodgkinia TETMLI1 RpoB fix 1	33	-23688.28048	Two ratio model	-19.753519	39.507038	1	3.26879E-10	
Hodgkinia TETMLI1 RpoB fix 1.2	33	-23693.68405	Two ratio model	-25.157082	50.314164	1	1.31001E-12	
Two ratio model Hodgkinia TETLIM1 RpoB	34	-23668.52696	One model	2.061149	4.122298	1	0.042321554	0.27923
Hodgkinia Tetuln RpoB fix 0.2	33	-23671.2369	Two ratio model	-2.001487	4.002974	1	0.045420054	
Hodgkinia Tetuln RpoB fix 1	33	-23749.87645	Two ratio model	-80.641043	161.282086	1	5.93656E-37	
Hodgkinia Tetuln RpoB fix 1.2	33	-23764.23757	Two ratio model	-95.002165	190.00433	1	3.17239E-43	
Two ratio model Hodgkinia Tetuln RpoB	34	-23669.23541	One model	1.352703	2.705406	1	0.100008619	0.14776
Hodgkinia Tetund1 RpoB fix 0.2	33	-23670.97033	Two ratio model	-1.867696	3.735392	1	0.053271166	
Hodgkinia Tetund1 RpoB fix 1	33	-23700.27901	Two ratio model	-31.176372	62.352744	1	2.87131E-15	
Hodgkinia Tetund1 RpoB fix 1.2	33	-23705.21976	Two ratio model	-36.117122	72.234244	1	1.9111E-17	
Two ratio model Hodgkinia Tetund1 RpoB	34	-23669.10264	One model	1.485476	2.970952	1	0.084771954	0.11596
Hodgkinia Tetund2 RpoB fix 0.2	33	-23670.18688	Two ratio model	-0.997479	1.994958	1	0.157823441	
Hodgkinia Tetund2 RpoB fix 1	33	-23680.80759	Two ratio model	-11.618188	23.236376	1	1.43262E-06	

Hodgkinia Tetund2 RpoB fix 1.2	33	-23684.10504	Two ratio model	-13.516923	27.033846	1	1.99924E-07	
Two ratio model Hodgkinia Tetund2 RpoB	34	-23669.1894	One model	1.398708	2.797416	1	0.094416359	0.28615
One Model Hodgkinia RpoC	63	-26023.04802	Free model	-66.701912	133.403824	30	5.3639E-15	
Free Model Hodgkinia RpoC	33	-26089.74993			0			
Hodgkinia Chocra RpoC Fix 0.2	33	-26092.44515	Two ratio model	3.241722	6.483444	1	0.010888381	
Hodgkinia Chocra RpoC Fix 1.0	33	-26118.57946	Two ratio model	-29.376035	58.75207	1	1.78845E-14	
Hodgkinia Chocra RpoC Fix 1.2	33	-26120.09827	Two ratio model	-30.894839	61.789678	1	3.82172E-15	
Two ratio Model Hodgkinia Chocra RpoC	34	-26089.20343	One model	0.546505	1.09301	1	0.295805251	0.13257
Hodgkinia DSEM RpoC Fix 0.2	33	-26099.38773	Two ratio model	-47.236077	94.472154	1	2.48565E-22	
Hodgkinia DSEM RpoC Fix 1.0	33	-26234.13505	Two ratio model	-181.983403	363.966806	1	3.85333E-81	
Hodgkinia DSEM RpoC Fix 1.2	33	-26254.99514	Two ratio model	-202.843487	405.686974	1	3.184E-90	
Two ratio Model Hodgkinia DSEM RpoC	34	-26052.15165	One model	37.598283	75.196566	1	4.26108E-18	0.01093
Hodgkinia TETAUR1a RpoC Fix 0.2	33	-26089.55992	Two ratio model	-0.176429	0.352858	1	0.552499727	
Hodgkinia TETAUR1a RpoC Fix 1.0	33	-26135.64325	Two ratio model	-46.25976	92.51952	1	6.6664E-22	
Hodgkinia TETAUR1a RpoC Fix 1.2	33	-26144.56209	Two ratio model	-55.178593	110.357186	1	8.18335E-26	
Two ratio Model Hodgkinia TETAUR1a RpoC	34	-26089.38349	One model	0.366441	0.732882	1	0.391950612	0.18029
Hodgkinia TETCHI1a RpoC Fix 0.2	33	-26090.22569	Two ratio model	-0.731602	1.463204	1	0.226421018	
Hodgkinia TETCHI1a RpoC Fix 1.0	33	-26099.3035	Two ratio model	-9.809409	19.618818	1	9.45335E-06	
Hodgkinia TETCHI1a RpoC Fix 1.2	33	-26100.73556	Two ratio model	-11.241472	22.482944	1	2.12018E-06	
Two ratio Model Hodgkinia TETCHI1a RpoC	34	-26089.49409	One model	0.255841	0.511682	1	0.474411636	0.10631
Hodgkinia TETCHI1b RpoC Fix 0.2	33	-26089.42102	Two ratio model	0.328912	0.657824	1	0.417329206	
Hodgkinia TETCHI1b RpoC Fix 1.0	33	-26092.58445	Two ratio model	-3.261579	6.523158	1	0.010647877	
Hodgkinia TETCHI1b RpoC Fix 1.2	33	-26093.36141	Two ratio model	-4.038536	8.077072	1	0.004482882	
Two ratio Model Hodgkinia TETCHI1b RpoC	34	-26089.32287	One model	0.427062	0.854124	1	0.35538829	0.25143
Hodgkinia TETCHI4 RpoC Fix 0.2	33	-26096.31705	Two ratio model	-11.228787	22.457574	1	2.14837E-06	
Hodgkinia TETCHI4 RpoC Fix 1.0	33	-26219.94298	Two ratio model	134.85471	269.70942	1	1.31293E-60	
Hodgkinia TETCHI4 RpoC Fix 1.2	33	-26240.86092	Two ratio model	155.772652	311.545304	1	1.00599E-69	

Two ratio Model Hodgkinia TETCHI4 RpoC	34	-26085.08827	One model	4.661668	9.323336	1	0.002262537	0.10092
Hodgkinia TETLIM2 RpoC Fix 0.2	33	-26086.90437	Two ratio model	-1.153491	2.306982	1	0.128793902	
Hodgkinia TETLIM2 RpoC Fix 1.0	33	-26091.79225	Two ratio model	-6.04137	12.08274	1	0.000508908	
Hodgkinia TETLIM2 RpoC Fix 1.2	33	-26092.51921	Two ratio model	-6.768325	13.53665	1	0.00023395	
Two ratio Model Hodgkinia TETLIM2 RpoC	34	-26085.75088	One model	3.999052	7.998104	1	0.004682636	0.27546
Hodgkinia TETLIM3 RpoC Fix 0.2	33	-26090.50754	Two ratio model	-0.870166	1.740332	1	0.187096766	
Hodgkinia TETLIM3 RpoC Fix 1.0	33	-26172.21886	Two ratio model	-82.58149	165.16298	1	8.42767E-38	
Hodgkinia TETLIM3 RpoC Fix 1.2	33	-26188.61714	Two ratio model	-98.979767	197.959534	1	5.82262E-45	
Two ratio Model Hodgkinia TETLIM3 RpoC	34	-26089.63737	One model	0.112562	0.225124	1	0.635163119	0.16604
Hodgkinia TETLIM4 RpoC Fix 0.2	33	-26090.66016	Two ratio model	-0.912658	1.825316	1	0.1766818	
Hodgkinia TETLIM4 RpoC Fix 1.0	33	-26144.81634	Two ratio model	-55.068837	110.137674	1	9.14161E-26	
Hodgkinia TETLIM4 RpoC Fix 1.2	33	-26155.10051	Two ratio model	-65.353008	130.706016	1	2.87131E-30	
Two ratio Model Hodgkinia TETLIM4 RpoC	34	-26089.74751	One model	0.002428	0.004856	1	0.944444377	0.15744
Hodgkinia TETLIM5 RpoC Fix 0.2	33	-26087.67433	Two ratio model	-0.076413	0.152826	1	0.695849229	
Hodgkinia TETLIM5 RpoC Fix 1.0	33	-26138.33536	Two ratio model	-50.737443	101.474886	1	7.23753E-24	
Hodgkinia TETLIM5 RpoC Fix 1.2	33	-26149.89357	Two ratio model	-62.29566	124.59132	1	6.2534E-29	
Two ratio Model Hodgkinia TETLIM5 RpoC	34	-26087.59791	One model	2.152019	4.304038	1	0.038021994	0.21232
Hodgkinia TETLON1 RpoC Fix 0.2	33	-26089.24672	Two ratio model	-0.000542	0.001084	1	0.97373507	
Hodgkinia TETLON1 RpoC Fix 1.0	33	-26111.01125	Two ratio model	-21.76507	43.53014	1	4.1748E-11	
Hodgkinia TETLON1 RpoC Fix 1.2	33	-26115.58163	Two ratio model	-26.33545	52.6709	1	3.94392E-13	
Two ratio Model Hodgkinia TETLON1 RpoC	34	-26089.24618	One model	0.503754	1.007508	1	0.315500586	0.19841
Hodgkinia TETLON2a RpoC Fix 0.2	33	-26089.33031	Two ratio model	-0.022318	0.044636	1	0.832674768	
Hodgkinia TETLON2a RpoC Fix 1.0	33	-26099.02948	Two ratio model	-9.721485	19.44297	1	1.03649E-05	
Hodgkinia TETLON2a RpoC Fix 1.2	33	-26101.1821	Two ratio model	-11.874099	23.748198	1	1.09798E-06	
Two ratio Model Hodgkinia TETLON2a RpoC	34	-26089.308	One model	0.441937	0.883874	1	0.347142813	0.21502
Hodgkinia TETLON2b RpoC Fix 0.2	33	-26088.74591	Two ratio model	-0.680732	1.361464	1	0.243283919	
Hodgkinia TETLON2b RpoC Fix 1.0	33	-26092.42261	Two ratio model	-4.357431	8.714862	1	0.003156263	

Hodgkinia TETLON2b RpoC Fix 1.2	33	-26093.68695	Two ratio model	-5.621766	11.243532	1	0.00079901	
Two ratio Model Hodgkinia TETLON2b RpoC	34	-26088.06518	One model	1.684754	3.369508	1	0.066413586	0.30978
Hodgkinia TETMLI1 RpoC Fix 0.2	33	-26090.7522	Two ratio model	-1.022493	2.044986	1	0.152708389	
Hodgkinia TETMLI1 RpoC Fix 1.0	33	-26132.9936	Two ratio model	-43.263893	86.527786	1	1.37788E-20	
Hodgkinia TETMLI1 RpoC Fix 1.2	33	-26140.66667	Two ratio model	-50.93697	101.87394	1	5.91698E-24	
Two ratio Model Hodgkinia TETMLI1 RpoC	34	-26089.7297	One model	0.020229	0.040458	1	0.840587744	0.14928
Hodgkinia TETULN RpoC Fix 0.2	33	-26092.84238	Two ratio model	-3.588203	7.176406	1	0.007386853	
Hodgkinia TETULN RpoC Fix 1.0	33	-26178.39548	Two ratio model	-89.141298	178.282596	1	1.14924E-40	
Hodgkinia TETULN RpoC Fix 1.2	33	-26192.64448	Two ratio model	-103.390306	206.780612	1	6.92262E-47	
Two ratio Model Hodgkinia TETULN RpoC	34	-26089.25418	One model	0.495756	0.991512	1	0.319373109	0.1347
Hodgkinia TETUND RpoC Fix 0.2	33	-26089.21715	Two ratio model	-0.000873	0.001746	1	0.966669961	
Hodgkinia TETUND RpoC Fix 1.0	33	-26113.41735	Two ratio model	-24.201066	48.402132	1	3.47191E-12	
Hodgkinia TETUND RpoC Fix 1.2	33	-26118.64396	Two ratio model	-29.427681	58.855362	1	1.69699E-14	
Two ratio Model Hodgkinia TETUND RpoC	34	-26089.21628	One model	0.533652	1.067304	1	0.301555203	0.19806
Hodgkinia TETUND2 RpoC Fix 0.2	33	-26089.31426	Two ratio model	-0.013131	0.026262	1	0.871262113	
Hodgkinia TETUND2 RpoC Fix 1.0	33	-26115.48503	Two ratio model	26.183904	52.367808	1	4.60206E-13	
Hodgkinia TETUND2 RpoC Fix 1.2	33	-26120.97819	Two ratio model	-31.677063	63.354126	1	1.72693E-15	
Two ratio Model Hodgkinia TETUND2 RpoC	34	-26089.30113	One model	0.448808	0.897616	1	0.343421754	0.19278
One Model Hodgkinia RpoD	63	-9295.347116	Free model	-376.347906	752.695812	30	4.8803E-139	
Free Model Hodgkinia RpoD	33	-9671.695022			0			
Hodgkinia CHOCRA RpoD Fix 0.2	33	-9661.196728	Two ratio model	0.682463	1.364926	1	0.242685584	
Hodgkinia CHOCRA RpoD Fix 1.0	33	-9695.741313	Two ratio model	35.227048	70.454096	1	4.71102E-17	
Hodgkinia CHOCRA RpoD Fix 1.2	33	-9705.707059	Two ratio model	45.192794	90.385588	1	1.95989E-21	
two ratio model hodgkinia CHOCRA RpoD	34	-9660.514265	One model	11.180757	22.361514	1	2.25855E-06	0.24937
Hodgkinia DSEM RpoD Fix 0.2	33	-9673.478005	Two ratio model	1.78386	3.56772	1	0.058913149	
Hodgkinia DSEM RpoD Fix 1.0	33	-9698.908904	Two ratio model	-27.214759	54.429518	1	1.61124E-13	
Hodgkinia DSEM RpoD Fix 1.2	33	-9704.656003	Two ratio model	32.961858	65.923716	1	4.68718E-16	

two ratio model hodgkinia DSEM RpoD	34	-9671.694145	One model	-0.000877	0.001754	1	0.966593736	0.08965
Hodgkinia TETAUR1a RpoD Fix 0.2	33	-9719.880537	Two ratio model	112.173312	224.346624	1	1.01933E-50	
Hodgkinia TETAUR1a RpoD Fix 1.0	33	-9895.094462	Two ratio model	287.387237	574.774474	1	5.1375E-127	
Hodgkinia TETAUR1a RpoD Fix 1.2	33	-9918.817104	Two ratio model	311.109879	622.219758	1	2.4602E-137	
two ratio model hodgkinia TETAUR1a RpoD	34	-9607.707225	One model	-63.987797	127.975594	1	1.13632E-29	0.00118
Hodgkinia TETCHI1a RpoD Fix 0.2	33	-9696.608589	Two ratio model	86.719082	173.438164	1	1.31305E-39	
Hodgkinia TETCHI1a RpoD Fix 1.0	33	-9717.512152	Two ratio model	107.622645	215.24529	1	9.85275E-49	
Hodgkinia TETCHI1a RpoD Fix 1.2	33	-9718.126878	Two ratio model	108.237371	216.474742	1	5.31325E-49	
two ratio model hodgkinia TETCHI1a RpoD	34	-9609.889507	One model	-61.805515	123.61103	1	1.02488E-28	0.00049
Hodgkinia TETCHI1b RpoD Fix 0.2	33	-9696.608589	Two ratio model	86.719158	173.438316	1	1.31295E-39	
Hodgkinia TETCHI1b RpoD Fix 1.0	33	-9717.512152	Two ratio model	107.622721	215.245442	1	9.852E-49	
Hodgkinia TETCHI1b RpoD Fix 1.2	33	-9718.126878	Two ratio model	108.237447	216.474894	1	5.31284E-49	
two ratio model hodgkinia TETCHI1b RpoD	34	-9609.889431	One model	61.805591	123.611182	1	1.0248E-28	0.00049
Hodgkinia TETCHI4 RpoD Fix 0.2	33	-9714.900878	Two ratio model	94.059905	188.11981	1	8.17987E-43	
Hodgkinia TETCHI4 RpoD Fix 1.0	33	-9873.740631	Two ratio model	252.899658	505.799316	1	5.202E-112	
Hodgkinia TETCHI4 RpoD Fix 1.2	33	-9895.380265	Two ratio model	274.539292	549.078584	1	1.9973E-121	
two ratio model hodgkinia TETCHI4 RpoD	34	-9620.840973	One model	50.854049	101.708098	1	6.43368E-24	0.00137
Hodgkinia TETLIM2 RpoD Fix 0.2	33	-9654.473642	Two ratio model	10.588557	21.177114	1	4.18735E-06	
Hodgkinia TETLIM2 RpoD Fix 1.0	33	-9643.989029	Two ratio model	-0.103944	0.207888	1	0.648427828	
Hodgkinia TETLIM2 RpoD Fix 1.2	33	-9643.911228	Two ratio model	-0.026143	0.052286	1	0.819132159	
two ratio model hodgkinia TETLIM2 RpoD	33	-9643.885085	One model	27.809937	55.619874	1	8.79302E-14	1.51062
Hodgkinia TETLIM3 RpoD Fix 0.2	33	-9661.117953	Two ratio model	3.185075	6.37015	1	0.011605579	
Hodgkinia TETLIM3 RpoD Fix 1.0	33	-9658.353038	Two ratio model	0.42016	0.84032	1	0.359305267	
Hodgkinia TETLIM3 RpoD Fix 1.2	33	-9658.512852	Two ratio model	0.579974	1.159948	1	0.281476325	
two ratio model hodgkinia TETLIM3 RpoD	34	-9657.932878	One model	13.762144	27.524288	1	1.55134E-07	0.50386
Hodgkinia TETLIM4 RpoD Fix 0.2	33	-9658.302756	Two ratio model	5.511902	11.023804	1	0.000899493	
Hodgkinia TETLIM4 RpoD Fix 1.0	33	-9654.324029	Two ratio model	1.533175	3.06635	1	0.079928746	

Hodgkinia TETLIM4 RpoD Fix 1.2	33	-9655.302661	Two ratio model	2.511807	5.023614	1	0.02500393	
two ratio model hodgkinia TETLIM4 RpoD	34	-9652.790854	One model	-18.904168	37.808336	1	7.80476E-10	0.55205
Hodgkinia TETLIM5 RpoD Fix 0.2	33	-9659.971783	Two ratio model	1.98392	3.96784	1	0.046377227	
Hodgkinia TETLIM5 RpoD Fix 1.0	33	-9665.264494	Two ratio model	7.276631	14.553262	1	0.000136253	
Hodgkinia TETLIM5 RpoD Fix 1.2	33	-9667.567087	Two ratio model	9.579224	19.158448	1	1.20304E-05	
two ratio model hodgkinia TETLIM5 RpoD	34	-9657.987863	One model	13.707159	27.414318	1	1.64211E-07	0.33727
Hodgkinia TETLON1 RpoD Fix 0.2	33	-9664.498748	Two ratio model	2.875291	5.750582	1	0.016483195	
Hodgkinia TETLON1 RpoD Fix 1.0	33	-9662.98611	Two ratio model	1.362653	2.725306	1	0.09876919	
Hodgkinia TETLON1 RpoD Fix 1.2	33	-9663.758924	Two ratio model	2.135467	4.270934	1	0.038769615	
two ratio model hodgkinia TETLON1 RpoD	34	-9661.623457	One model	10.071565	20.14313	1	7.18579E-06	0.50796
Hodgkinia TETLON2a RpoD Fix 0.2	33	-9670.553966	Two ratio model	0.023795	0.04759	1	0.82731121	
Hodgkinia TETLON2a RpoD Fix 1.0	33	-9673.35815	Two ratio model	2.827979	5.655958	1	0.017396169	
Hodgkinia TETLON2a RpoD Fix 1.2	33	-9673.995931	Two ratio model	3.46576	6.93152	1	0.008468968	
two ratio model hodgkinia TETLON2a RpoD	34	-9670.530171	One model	1.164851	2.329702	1	0.126926174	0.22805
Hodgkinia TETLON2b RpoD Fix 0.2	33	-9671.496969	Two ratio model	0.071765	0.14353	1	0.704796631	
Hodgkinia TETLON2b RpoD Fix 1.0	33	-9674.962674	Two ratio model	3.53747	7.07494	1	0.007816944	
Hodgkinia TETLON2b RpoD Fix 1.2	33	-9675.600346	Two ratio model	4.175142	8.350284	1	0.003856271	
two ratio model hodgkinia TETLON2b RpoD	34	-9671.425204	One model	0.269818	0.539636	1	0.462583619	0.15279
Hodgkinia TETMLI1 RpoD Fix 0.2	33	-9663.498138	Two ratio model	-1.470185	2.94037	1	0.086390942	
Hodgkinia TETMLI1 RpoD Fix 1.0	33	-9667.397922	Two ratio model	5.369969	10.739938	1	0.001048478	
Hodgkinia TETMLI1 RpoD Fix 1.2	33	-9669.113457	Two ratio model	7.085504	14.171008	1	0.000166923	
two ratio model hodgkinia TETMLI1 RpoD	34	-9662.027953	One model	9.667069	19.334138	1	1.09727E-05	0.33929
Hodgkinia Tetuln RpoD Fix 0.2	33	-9659.216048	Two ratio model	1.503048	3.006096	1	0.082951858	
Hodgkinia Tetuln RpoD Fix 1.0	33	-9666.855254	Two ratio model	9.142254	18.284508	1	1.90248E-05	
Hodgkinia Tetuln RpoD Fix 1.2	33	-9669.12902	Two ratio model	11.41602	22.83204	1	1.76794E-06	
two ratio model hodgkinia Tetuln RpoD	34	-9657.713	One model	13.982022	27.964044	1	1.23591E-07	0.30496
Hodgkinia Tetund1 RpoD Fix 0.2	33	-9670.982347	Two ratio model	0.108662	0.217324	1	0.641086505	

Hodgkinia Tetund1 RpoD Fix 1.0	33	-9679.577683	Two ratio model	8.703998	17.407996	1	3.01555E-05	
Hodgkinia Tetund1 RpoD Fix 1.2	33	-9681.297173	Two ratio model	10.423488	20.846976	1	4.97477E-06	
two ratio model hodgkinia Tetund1 RpoD	34	-9670.873685	One model	0.821337	1.642674	1	0.199958979	0.16194
Hodgkinia Tetund2 RpoD Fix 0.2	33	-9668.539366	Two ratio model	0.108363	0.216726	1	0.641545946	
Hodgkinia Tetund2 RpoD Fix 1.0	33	-9675.219614	Two ratio model	6.788611	13.577222	1	0.000228947	
Hodgkinia Tetund2 RpoD Fix 1.2	33	-9676.895286	Two ratio model	8.464283	16.928566	1	3.88131E-05	
two ratio model hodgkinia Tetund2 RpoD	34	-9668.431003	One model	3.264019	6.528038	1	0.010618702	0.23894
One model Tremblaya RpoA	15	-2628.650599	Free model	-10.82393	21.64786	12	0.041661278	
Free model Tremblaya RpoA	27	-2617.826669						
Tremblaya PAVE RpoA fix 0.2	15	-2628.242309	Two ratio model	2.849086	5.698172	1	0.016982591	
Tremblaya PAVE RpoA fix 1	15	-2649.218015	Two ratio model	23.824792	47.649584	1	5.09631E-12	
Tremblaya PAVE RpoA fix 1.2	15	-2653.191702	Two ratio model	27.798479	55.596958	1	8.89612E-14	
Two ratio modelTremblaya PAVE RpoA	16	-2625.393223	One model	-3.257376	6.514752	1	0.010698324	0.01543
Tremblaya PCIT RpoA fix 0.2	15	-2628.650599	Two ratio model	-6E-06	1.2E-05	1	0.997236052	
Tremblaya PCIT RpoA fix 1	15	-2628.650595	Two ratio model	2E-06	4E-06	1	0.998404232	
Tremblaya PCIT RpoA fix 1.2	15	-2628.650595	Two ratio model	2E-06	4E-06	1	0.998404232	
Two ratio model Tremblaya PCIT RpoA	16	-2628.650593	One model	-6E-06	1.2E-05	1	0.997236052	10.09443
Tremblaya PCVAL RpoA fix 0.2	15	-2628.650599	Two ratio model	-7E-06	1.4E-05	1	0.997014596	
Tremblaya PCVAL RpoA fix 1	15	-2628.650595	Two ratio model	-3E-06	6E-06	1	0.998045592	
Tremblaya PCVAL RpoA fix 1.2	15	-2628.650595	Two ratio model	-3E-06	6E-06	1	0.998045592	
Two ratio model Tremblaya PCVAL RpoA	16	-2628.650592	One model	-7E-06	1.4E-05	1	0.997014596	103.23119
Tremblaya TPFVIR RpoA fix 0.2	15	-2628.659117	Two ratio model	-0.970195	1.94039	1	0.163626195	
Tremblaya TPFVIR RpoA fix 1	15	-2632.189297	Two ratio model	-4.500375	9.00075	1	0.002698688	
Tremblaya TPFVIR RpoA fix 1.2	15	-2633.71236	Two ratio model	-6.023438	12.046876	1	0.000518792	
Two ratio model Tremblaya TPFVIR RpoA	16	-2627.688922	One model	-0.961677	1.923354	1	0.165487388	0.33268
Tremblaya TPMHIR1 RpoA fix 0.2	15	-2628.619516	Two ratio model	-0.060612	0.121224	1	0.727710736	
Tremblaya TPMHIR1 RpoA fix 1	15	-2629.986392	Two ratio model	-1.427488	2.854976	1	0.091091599	

Tremblaya TPMHIR1 RpoA fix 1.2	15	-2630.044429	Two ratio model	-1.485525	2.97105	1	0.084766819	
Two ratio model Tremblaya TPMHIR1 RpoA	16	-2628.558904	One model	-0.091695	0.18339	1	0.66847597	0.16851
Tremblaya TPPLON1 RpoA fix 0.2	15	-2628.64141	Two ratio model	2.600605	5.20121	1	0.022571171	
Tremblaya TPPLON1 RpoA fix 1	15	-2626.285638	Two ratio model	0.244833	0.489666	1	0.484076332	
Tremblaya TPPLON1 RpoA fix 1.2	15	-2626.541002	Two ratio model	0.500197	1.000394	1	0.31721519	
Two ratio model Tremblaya TPPLON1 RpoA	16	-2626.040805	One model	-2.609794	5.219588	1	0.022333842	0.67027
Tremblaya TPPMAR RpoA fix 0.2	15	-2628.618007	Two ratio model	-0.104931	0.209862	1	0.646875588	
Tremblaya TPPMAR RpoA fix 1	15	-2633.176067	Two ratio model	4.662991	9.325982	1	0.002259272	
Tremblaya TPPMAR RpoA fix 1.2	15	-2634.044953	Two ratio model	-5.531877	11.063754	1	0.000880317	
Two ratio model Tremblaya TPPMAR RpoA	16	-2628.513076	One model	-0.137523	0.275046	1	0.599966783	0.14871
Tremblaya TPTPER RpoA fix 0.2	15	-2628.631513	Two ratio model	-0.011617	0.023234	1	0.878850138	
Tremblaya TPTPER RpoA fix 1	15	-2636.93901	Two ratio model	-8.319114	16.638228	1	4.52301E-05	
Tremblaya TPTPER RpoA fix 1.2	15	-2638.756655	Two ratio model	-10.136759	20.273518	1	6.71232E-06	
Two ratio model Tremblaya TPTPER RpoA	16	-2628.619896	One model	-0.030703	0.061406	1	0.804287344	0.18754
One model Tremblaya RpoB	15	-15984.03205	Free model	-24.910205	49.82041	12	1.50233E-06	
Free model Tremblaya RpoB	27	-15959.12185						
Tremblaya PAVE RpoB fix 0.2	15	-15984.62366	Two ratio model	20.23252	40.46504	1	2.00166E-10	
Tremblaya PAVE RpoB fix 1	15	-16105.98535	Two ratio model	141.59421	283.18842	1	1.51634E-63	
Tremblaya PAVE RpoB fix 1.2	15	-16127.88284	Two ratio model	163.4917	326.9834	1	4.36331E-73	
Two ratio modelTremblaya PAVE RpoB	16	-15964.39114	One model	-19.640912	39.281824	1	3.6684E-10	0.01134
Tremblaya PCIT RpoB fix 0.2	15	-15983.79212	Two ratio model	0.18533	0.37066	1	0.542644731	
Tremblaya PCIT RpoB fix 1	15	-16005.61957	Two ratio model	22.01278	44.02556	1	3.24116E-11	
Tremblaya PCIT RpoB fix 1.2	15	-16010.74881	Two ratio model	27.14202	54.28404	1	1.73505E-13	
Two ratio model Tremblaya PCIT RpoB	16	-15983.60679	One model	0.425262	0.850524	1	0.356404143	0.22825
Tremblaya PCVAL RpoB fix 0.2	15	-15984.03205	Two ratio model	-1E-04	0.0002	1	0.988716584	
Tremblaya PCVAL RpoB fix 1	15	-15984.03211	Two ratio model	-4E-05	8E-05	1	0.992863599	
Tremblaya PCVAL RpoB fix 1.2	15	-15984.03212	Two ratio model	-3E-05	6E-05	1	0.993819675	

Two ratio model Tremblaya PCVAL RpoB	16	-15984.03215	One model	-9.8E-05	0.000196	1	0.988829981	34.176
Tremblaya TPFVIR RpoB fix 0.2	15	-15983.8562	Two ratio model	0.02295	0.0459	1	0.83035768	
Tremblaya TPFVIR RpoB fix 1	15	-16029.83463	Two ratio model	46.00138	92.00276	1	8.65557E-22	
Tremblaya TPFVIR RpoB fix 1.2	15	-16040.15323	Two ratio model	56.31998	112.63996	1	2.5874E-26	
Two ratio model Tremblaya TPFVIR RpoB	16	-15983.83325	One model	0.198802	0.397604	1	0.528329252	0.20709
Tremblaya TPMHIR1 RpoB fix 0.2	15	-15984.17104	Two ratio model	0.14624	0.29248	1	0.588635654	
Tremblaya TPMHIR1 RpoB fix 1	15	-16004.00473	Two ratio model	19.97993	39.95986	1	2.59236E-10	
Tremblaya TPMHIR1 RpoB fix 1.2	15	-16005.28699	Two ratio model	21.26219	42.52438	1	6.98067E-11	
Two ratio model Tremblaya TPMHIR1 RpoB	16	-15984.0248	One model	0.007252	0.014504	1	0.904140505	0.18484
Tremblaya TPPLON1 RpoB fix 0.2	15	-15983.79212	Two ratio model	0.18533	0.37066	1	0.542644731	
Tremblaya TPPLON1 RpoB fix 1	15	-16005.61957	Two ratio model	22.01278	44.02556	1	3.24116E-11	
Tremblaya TPPLON1 RpoB fix 1.2	15	-16010.74881	Two ratio model	27.14202	54.28404	1	1.73505E-13	
Two ratio model Tremblaya TPPLON1 RpoB	16	-15983.60679	One model	0.425262	0.850524	1	0.356404143	0.22825
Tremblaya TPPMAR RpoB fix 0.2	15	-15983.79288	Two ratio model	0.12098	0.24196	1	0.62279407	
Tremblaya TPPMAR RpoB fix 1	15	-16012.28718	Two ratio model	28.61528	57.23056	1	3.87601E-14	
Tremblaya TPPMAR RpoB fix 1.2	15	-16018.5231	Two ratio model	34.8512	69.7024	1	6.89621E-17	
Two ratio model Tremblaya TPPMAR RpoB	16	-15983.6719	One model	0.360152	0.720304	1	0.39604421	0.21979
Tremblaya TPTPER RpoB fix 0.2	15	-15984.20801	Two ratio model	0.21047	0.42094	1	0.516468375	
Tremblaya TPTPER RpoB fix 1	15	-16042.63604	Two ratio model	58.6385	117.277	1	2.49649E-27	
Tremblaya TPTPER RpoB fix 1.2	15	-16054.4592	Two ratio model	70.46166	140.92332	1	1.67228E-32	
Two ratio model Tremblaya TPTPER RpoB	16	-15983.99754	One model	0.034512	0.069024	1	0.792763171	0.18072
One model Tremblaya RpoC	15	-15773.42764	Free model	-25.974692	51.949384	12	6.32904E-07	
Free model Tremblaya RpoC	27	-15747.45295						
Tremblaya PAVE RpoC fix 0.2	15	-15786.70169	Two ratio model	-33.7661	67.5322	1	2.07274E-16	
Tremblaya PAVE RpoC fix 1	15	-15968.58114	Two ratio model	-215.645546	431.291092	1	8.50909E-96	
Tremblaya PAVE RpoC fix 1.2	15	-15997.5242	Two ratio model	-244.588603	489.177206	1	2.152E-108	
Two ratio modelTremblaya PAVE RpoC	16	-15752.93559	One model	20.492051	40.984102	1	1.53473E-10	0.00928

Tremblaya PCIT RpoC fix 0.2	15	-15773.42766	Two ratio model	0.000147	0.000294	1	0.9863198	
Tremblaya PCIT RpoC fix 1	15	-15773.42775	Two ratio model	6.1E-05	0.000122	1	0.991187256	
Tremblaya PCIT RpoC fix 1.2	15	-15773.42775	Two ratio model	5.3E-05	0.000106	1	0.991785421	
Two ratio model Tremblaya PCIT RpoC	16	-15773.42781	One model	-0.000163	0.000326	1	0.985594602	40.84519
Tremblaya PCVAL RpoC fix 0.2	15	-15773.42766	Two ratio model	0.000133	0.000266	1	0.986987474	
Tremblaya PCVAL RpoC fix 1	15	-15773.42775	Two ratio model	4.7E-05	9.4E-05	1	0.992264343	
Tremblaya PCVAL RpoC fix 1.2	15	-15773.42775	Two ratio model	3.9E-05	7.8E-05	1	0.992953366	
Two ratio model Tremblaya PCVAL RpoC	16	-15773.42779	One model	-0.000149	0.000298	1	0.986227061	5.21702
Tremblaya TPFVIR RpoC fix 0.2	15	-15773.28037	Two ratio model	-0.21724	0.43448	1	0.509798829	
Tremblaya TPFVIR RpoC fix 1	15	-15791.60376	Two ratio model	-18.54063	37.08126	1	1.13307E-09	
Tremblaya TPFVIR RpoC fix 1.2	15	-15793.29819	Two ratio model	-20.235058	40.470116	1	1.99646E-10	
Two ratio model Tremblaya TPFVIR RpoC	16	-15773.06313	One model	0.36451	0.72902	1	0.393201029	0.17192
Tremblaya TPMHIR1 RpoC fix 0.2	15	-15770.75127	Two ratio model	-0.000656	0.001312	1	0.97110571	
Tremblaya TPMHIR1 RpoC fix 1	15	-15781.43895	Two ratio model	-10.688337	21.376674	1	3.77334E-06	
Tremblaya TPMHIR1 RpoC fix 1.2	15	-15782.25154	Two ratio model	-11.500933	23.001866	1	1.61844E-06	
Two ratio model Tremblaya TPMHIR1 RpoC	16	-15770.75061	One model	2.677033	5.354066	1	0.020674005	0.19874
Tremblaya TPPLON1 RpoC fix 0.2	15	-15773.72983	Two ratio model	-0.440663	0.881326	1	0.347838756	
Tremblaya TPPLON1 RpoC fix 1	15	-15803.66916	Two ratio model	-30.379995	60.75999	1	6.44751E-15	
Tremblaya TPPLON1 RpoC fix 1.2	15	-15809.44466	Two ratio model	-36.155488	72.310976	1	1.83822E-17	
Two ratio model Tremblaya TPPLON1 RpoC	16	-15773.28917	One model	0.138474	0.276948	1	0.598708619	0.16033
Tremblaya TPPMAR RpoC fix 0.2	15	-15774.31884	Two ratio model	-0.896409	1.792818	1	0.180583194	
Tremblaya TPPMAR RpoC fix 1	15	-15805.9353	Two ratio model	-32.512866	65.025732	1	7.39264E-16	
Tremblaya TPPMAR RpoC fix 1.2	15	-15811.57787	Two ratio model	-38.155431	76.310862	1	2.42347E-18	
Two ratio model Tremblaya TPPMAR RpoC	16	-15773.42244	One model	0.005208	0.010416	1	0.918709998	0.14528
Tremblaya TPTPER RpoC fix 0.2	15	-15771.79292	Two ratio model	-0.021496	0.042992	1	0.835740337	
Tremblaya TPTPER RpoC fix 1	15	-15815.46133	Two ratio model	-43.689907	87.379814	1	8.95599E-21	
Tremblaya TPTPER RpoC fix 1.2	15	-15824.79874	Two ratio model	-53.02732	106.05464	1	7.17299E-25	

Two ratio model Tremblaya TPTPER RpoC	16	-15771.77142	One model	1.656221	3.312442	1	0.068757248	0.19287
One model Tremblaya RpoD	15	-3895.383633	Free model	-6.31495	12.6299	12	0.396504813	
Free nidek Tremblaya RpoD	27	-3889.068683						
Tremblaya PAVE RpoD fix 0.2	15	-3895.757264	Two ratio model	3.579402	7.158804	1	0.007459692	
Tremblaya PAVE RpoD fix 1	15	-3917.573302	Two ratio model	25.39544	50.79088	1	1.0275E-12	
Tremblaya PAVE RpoD fix 1.2	15	-3923.133773	Two ratio model	30.955911	61.911822	1	3.59187E-15	
Two ratio modelTremblaya PAVE RpoD	16	-3892.177862	One model	-3.205771	6.411542	1	0.011338091	0.01485
Tremblaya PCIT RpoD fix 0.2	15	-3895.383634	Two ratio model	9E-06	1.8E-05	1	0.996614873	
Tremblaya PCIT RpoD fix 1	15	-3895.383645	Two ratio model	2E-05	4E-05	1	0.994953769	
Tremblaya PCIT RpoD fix 1.2	15	-3895.383646	Two ratio model	2.1E-05	4.2E-05	1	0.994829153	
Two ratio model Tremblaya PCIT RpoD	16	-3895.383625	One model	-8E-06	1.6E-05	1	0.99680847	0.0001
Tremblaya PCVAL RpoD fix 0.2	15	-3895.383634	Two ratio model	-1.3E-05	2.6E-05	1	0.995931589	
Tremblaya PCVAL RpoD fix 1	15	-3895.383645	Two ratio model	-2E-06	4E-06	1	0.998404232	
Tremblaya PCVAL RpoD fix 1.2	15	-3895.383646	Two ratio model	-1E-06	2E-06	1	0.998871621	
Two ratio model Tremblaya PCVAL RpoD	16	-3895.383647	One model	1.4E-05	2.8E-05	1	0.995778011	1.51337
Tremblaya TPFVIR RpoD fix 0.2	15	-3894.979864	Two ratio model	0.418198	0.836396	1	0.360429555	
Tremblaya TPFVIR RpoD fix 1	15	-3898.545208	Two ratio model	3.983542	7.967084	1	0.004763562	
Tremblaya TPFVIR RpoD fix 1.2	15	-3899.158823	Two ratio model	4.597157	9.194314	1	0.002427681	
Two ratio model Tremblaya TPFVIR RpoD	16	-3894.561666	One model	-0.821967	1.643934	1	0.199786561	0.28058
Tremblaya TPMHIR1 RpoD fix 0.2	15	-3895.852801	Two ratio model	0.77684	1.55368	1	0.212592994	
Tremblaya TPMHIR1 RpoD fix 1	15	-3953.896721	Two ratio model	58.82076	117.64152	1	2.07736E-27	
Tremblaya TPMHIR1 RpoD fix 1.2	15	-3965.996133	Two ratio model	70.920172	141.840344	1	1.05388E-32	
Two ratio model Tremblaya TPMHIR1 RpoD	16	-3895.075961	One model	-0.307672	0.615344	1	0.432782765	0.28058
Tremblaya TPPLON1 RpoD fix 0.2	15	-3894.98117	Two ratio model	0.335202	0.670404	1	0.41291079	
Tremblaya TPPLON1 RpoD fix 1	15	-3901.690501	Two ratio model	7.044533	14.089066	1	0.000174354	
Tremblaya TPPLON1 RpoD fix 1.2	15	-3903.572074	Two ratio model	8.926106	17.852212	1	2.38742E-05	
Two ratio model Tremblaya TPPLON1 RpoD	16	-3894.645968	One model	-0.737665	1.47533	1	0.224506619	0.26544

Tremblaya TPPMAR RpoD fix 0.2	15	-3895.587147	Two ratio model	1.010858	2.021716	1	0.15506382	
Tremblaya TPPMAR RpoD fix 1	15	-3901.716838	Two ratio model	7.140549	14.281098	1	0.000157438	
Tremblaya TPPMAR RpoD fix 1.2	15	-3902.588947	Two ratio model	8.012658	16.025316	1	6.25011E-05	
Two ratio model Tremblaya TPPMAR RpoD	16	-3894.576289	One model	-0.807344	1.614688	1	0.203834064	0.07076
Tremblaya TPTPER RpoD fix 0.2	15	-3895.389201	Two ratio model	0.006941	0.013882	1	0.906208777	
Tremblaya TPTPER RpoD fix 1	15	-3896.016757	Two ratio model	0.634497	1.268994	1	0.25995549	
Tremblaya TPTPER RpoD fix 1.2	15	-3896.05124	Two ratio model	0.66898	1.33796	1	0.247394047	
Two ratio model Tremblaya TPTPER RpoD	16	-3895.38226	One model	-0.001373	0.002746	1	0.958208149	0.18448
One model carsonella RpoA	17	-4306.896555	Free model	-7.227934	14.455868	14	0.416329801	0.00436
Free Model carsonella RpoA	31	-4299.668621						
Carsonella BC RpoA Fix 0.2	17	-4308.596342	Two ratio model	1.717554	3.435108	1	0.063824412	
Carsonella BC RpoA Fix 1.0	17	-4309.052484	Two ratio model	2.173696	4.347392	1	0.037065665	
Carsonella BC RpoA Fix 1.2	17	-4309.079591	Two ratio model	2.173696	4.347392	1	0.037065665	
Two ratio model carsonella BC RpoA	18	-4306.878788	One model	0.017767	0.035534	1	0.850481077	0.0032
Carsonella CE RpoA Fix 0.2	17	-4301.697008	Two ratio model	-0.105822	0.211644	1	0.645481894	
Carsonella CE RpoA Fix 1.0	17	-4301.586459	Two ratio model	-0.216371	0.432742	1	0.510646549	
Carsonella CE RpoA Fix 1.2	17	-4301.581011	Two ratio model	-0.221819	0.443638	1	0.505371754	
Two ratio model carsonella CE RpoA	18	-4301.80283	One model	5.093725	10.18745	1	0.001413997	13.9
Carsonella CS RpoA Fix 0.2	17	-4304.625473	Two ratio model	0.073783	0.147566	1	0.700872312	
Carsonella CS RpoA Fix 1.0	17	-4304.566356	Two ratio model	0.014666	0.029332	1	0.864014712	
Carsonella CS RpoA Fix 1.2	17	-4304.563903	Two ratio model	0.012213	0.024426	1	0.875805861	
Two ratio model carsonella CS RpoA	18	-4304.55169	One model	2.344865	4.68973	1	0.030343415	3
Carsonella DC RpoA Fix 0.2	17	-4306.896615	Two ratio model	4.5E-05	9E-05	1	0.992430716	
Carsonella DC RpoA Fix 1.0	17	-4306.896664	Two ratio model	9.4E-05	0.000188	1	0.989060301	
Carsonella DC RpoA Fix 1.2	17	-4306.896678	Two ratio model	0.000108	0.000216	1	0.988273962	
Two ratio model carsonella DC RpoA	18	-4306.89657	One model	-1.5E-05	3E-05	1	0.995629828	0.0001
Carsonella HC RpoA Fix 0.2	17	-4309.698626	Two ratio model	2.874316	5.748632	1	0.016501502	

Carsonella HC RpoA Fix 1.0	17	-4310.394521	Two ratio model	3.570211	7.140422	1	0.007536542	
Carsonella HC RpoA Fix 1.2	17	-4310.430967	Two ratio model	3.606657	7.213314	1	0.007236476	
Two ratio model carsonella HC RpoA	18	-4306.82431	One model	0.072245	0.14449	1	0.703857526	0.00268
Carsonella HT RpoA Fix 0.2	17	-4307.418221	Two ratio model	0.52637	1.05274	1	0.304876883	
Carsonella HT RpoA Fix 1.0	17	-4307.582458	Two ratio model	0.690607	1.381214	1	0.239894467	
Carsonella HT RpoA Fix 1.2	17	-4307.591899	Two ratio model	0.700048	1.400096	1	0.236707498	
Two ratio model carsonella HT RpoA	18	-4306.891851	One model	0.004704	0.009408	1	0.922730488	0.0037
Carsonella PC RpoA Fix 0.2	17	-4310.177886	Two ratio model	7.265566	14.531132	1	0.000137862	
Carsonella PC RpoA Fix 1.0	17	-4310.567752	Two ratio model	7.655432	15.310864	1	9.11906E-05	
Carsonella PC RpoA Fix 1.2	17	-4310.59401	Two ratio model	7.68169	15.36338	1	8.86906E-05	
Two ratio model carsonella PC RpoA	18	-4302.91232	One model	3.984235	7.96847	1	0.004759915	0.00042
Carsonella PV RpoA Fix 0.2	17	-4308.57644	Two ratio model	5.225614	10.451228	1	0.001225679	
Carsonella PV RpoA Fix 1.0	17	-4308.642881	Two ratio model	5.292055	10.58411	1	0.001140638	
Carsonella PV RpoA Fix 1.2	17	-4308.64594	Two ratio model	5.295114	10.590228	1	0.00113687	
Two ratio model carsonella PV RpoA	18	-4303.350826	One model	3.545729	7.091458	1	0.007745221	0.00027
Carsonella YCCR RpoA Fix 0.2	17	-4306.896627	Two ratio model	0.0001	0.0002	1	0.988716584	
Carsonella YCCR RpoA Fix 1.0	17	-4306.896623	Two ratio model	9.6E-05	0.000192	1	0.988944541	
Carsonella YCCR RpoA Fix 1.2	17	-4306.896656	Two ratio model	0.000129	0.000258	1	0.987184627	
Two ratio model carsonella YCCR RpoA	18	-4306.896527	One model	2.8E-05	5.6E-05	1	0.994029234	0.0001
One model carsonella RpoB	17	-16338.12467	Free model	-10.708737	21.417474	14	0.091402783	0.00828
Free Model Carsonella RpoB	31	-16327.41593						
Carsonella BC RpoB Fix 0.2	17	-16352.53475	Two ratio model	11.953318	23.906636	1	1.01123E-06	
Carsonella BC RpoB Fix 1.0	17	-16362.375	Two ratio model	21.793567	43.587134	1	4.05496E-11	
Carsonella BC RpoB Fix 1.2	17	-16363.105	Two ratio model	22.523564	45.047128	1	1.92349E-11	
Two ratio model carsonella BC RpoB	18	-16340.58143	One model	-2.456766	4.913532	1	0.026647099	0.00049
Carsonella CE RpoB Fix 0.2	17	-16349.11096	Two ratio model	-11.631839	23.263678	1	1.41242E-06	
Carsonella CE RpoB Fix 1.0	17	-16350.43612	Two ratio model	12.956993	25.913986	1	3.56973E-07	

Carsonella CE RpoB Fix 1.2	17	-16350.49977	Two ratio model	13.020644	26.041288	1	3.34193E-07	
Two rati model carsonella CE RpoB	18	-16337.47913	One model	0.645539	1.291078	1	0.255849384	0.0058
Carsonella CS RpoB Fix 0.2	17	-16340.41108	Two ratio model	2.311015	4.62203	1	0.031563862	
Carsonella CS RpoB Fix 1.0	17	-16340.78604	Two ratio model	-2.685966	5.371932	1	0.0204633	
Carsonella CS RpoB Fix 1.2	17	-16340.80409	Two ratio model	2.704021	5.408042	1	0.020044186	
Two rati model carsonella CS RpoB	18	-16338.10007	One model	0.024596	0.049192	1	0.824475266	0.00725
Carsonella DC RpoB Fix 0.2	17	-16338.12584	Two ratio model	-0.001912	0.003824	1	0.950691444	
Carsonella DC RpoB Fix 1.0	17	-16338.12654	Two ratio model	3.6E-05	7.2E-05	1	0.993229806	
Carsonella DC RpoB Fix 1.2	17	-16338.12644	Two ratio model	0.000134	0.000268	1	0.98693865	
Two rati model carsonella DC RpoB	18	-16338.12658	One model	-0.001912	0.003824	1	0.950691444	4.02786
Carsonella HC RpoB Fix 0.2	17	-16340.66086	Two ratio model	2.591162	5.182324	1	0.022817785	
Carsonella HC RpoB Fix 1.0	17	-16341.35343	Two ratio model	3.283732	6.567464	1	0.010385984	
Carsonella HC RpoB Fix 1.2	17	-16341.38904	Two ratio model	3.319343	6.638686	1	0.009978751	
Two rati model carsonella HC RpoB	18	-16338.0697	One model	0.054967	0.109934	1	0.740219288	0.01017
Carsonella HT RpoB Fix 0.2	17	-16354.3356	Two ratio model	17.676252	35.352504	1	2.75115E-09	
Carsonella HT RpoB Fix 1.0	17	-16357.63307	Two ratio model	20.973718	41.947436	1	9.37602E-11	
Carsonella HT RpoB Fix 1.2	17	-16357.81799	Two ratio model	21.158646	42.317292	1	7.76034E-11	
Two rati model carsonella HT RpoB	18	-16336.65935	One model	1.465318	2.930636	1	0.086913247	0.0045
Carsonella PC RpoB Fix 0.2	17	-16344.15722	Two ratio model	6.131388	12.262776	1	0.000462084	
Carsonella PC RpoB Fix 1.0	17	-16346.16239	Two ratio model	8.13656	16.27312	1	5.48364E-05	
Carsonella PC RpoB Fix 1.2	17	-16346.27326	Two ratio model	8.247428	16.494856	1	4.87822E-05	
Two rati model carsonella PC RpoB	18	-16338.02583	One model	0.098835	0.19767	1	0.656608165	0.01018
Carsonella PV RpoB Fix 0.2	17	-16338.57381	Two ratio model	2.414283	4.828566	1	0.027991904	
Carsonella PV RpoB Fix 1.0	17	-16340.37792	Two ratio model	4.218389	8.436778	1	0.003677072	
Carsonella PV RpoB Fix 1.2	17	-16340.48592	Two ratio model	4.326387	8.652774	1	0.003265638	
Two rati model carsonella PV RpoB	18	-16336.15953	One model	1.965134	3.930268	1	0.047424386	0.02498
Carsonella YCCR RpoB Fix 0.2	17	-16337.66901	Two ratio model	0.612337	1.224674	1	0.268445324	

Carsonella YCCR RpoB Fix 1.0	17	-16337.69745	Two ratio model	0.185117	0.370234	1	0.542876746	
Carsonella YCCR RpoB Fix 1.2	17	-16337.69861	Two ratio model	-0.18628	0.37256	1	0.541612145	
Two rati model carsonella YCCR RpoB	18	-16337.51233	One model	0.612337	1.224674	1	0.268445324	0.03572
One Model RpoC Carsonellas	17	-16436.95241	Free model	-9.879244	19.758488	14	0.13794148	
Free Model Carsonella RpoC	31	-16427.07317						
Carsonella BC RpoC fix 0.2	17	-16461.3104	Two ratio model	-25.21795	50.4359	1	1.23121E-12	
Carsonella BC RpoC fix 1	17	-16473.2557	Two ratio model	-37.16325	74.3265	1	6.62087E-18	
Carsonella BC RpoC fix 1.2	17	-16474.11487	Two ratio model	-38.02242	76.04484	1	2.77296E-18	
Two ratio model Carsonella BC RpoC	18	-16436.09245	One model	0.859963	1.719926	1	0.189702571	0.00952
Carsonella CE RpoC fix 0.2	17	-16444.31465	Two ratio model	8.31653	16.63306	1	4.53536E-05	
Carsonella CE RpoC fix 1	17	-16445.13911	Two ratio model	9.14099	18.28198	1	1.90501E-05	
Carsonella CE RpoC fix 1.2	17	-16445.17459	Two ratio model	-9.17647	18.35294	1	1.83536E-05	
Two ratio model Carsonella CE RpoC	18	-16435.99812	One model	0.954293	1.908586	1	0.167120413	0.00871
Carsonella CS RpoC fix 0.2	17	-16443.46076	Two ratio model	-7.13149	14.26298	1	0.000158961	
Carsonella CS RpoC fix 1	17	-16444.38678	Two ratio model	8.05751	16.11502	1	5.96094E-05	
Carsonella CS RpoC fix 1.2	17	-16444.42906	Two ratio model	-8.09979	16.19958	1	5.70068E-05	
Two ratio model Carsonella CS RpoC	18	-16436.32927	One model	0.623143	1.246286	1	0.264263018	0.00939
Carsonella DC RpoC fix 0.2	17	-16436.95996	Two ratio model	0.12607	0.25214	1	0.615572252	
Carsonella DC RpoC fix 1	17	-16436.96144	Two ratio model	0.12755	0.2551	1	0.613506685	
Carsonella DC RpoC fix 1.2	17	-16436.96153	Two ratio model	0.12764	0.25528	1	0.613381562	
Two ratio model Carsonella DC RpoC	18	-16436.83389	One model	0.118523	0.237046	1	0.626347838	0.0001
Carsonella HC RpoC fix 0.2	17	-16447.50503	Two ratio model	11.12826	22.25652	1	2.38547E-06	
Carsonella HC RpoC fix 1	17	-16450.42801	Two ratio model	14.05124	28.10248	1	1.15058E-07	
Carsonella HC RpoC fix 1.2	17	-16450.59855	Two ratio model	14.22178	28.44356	1	9.64701E-08	
Two ratio model Carsonella HC RpoC	18	-16436.37677	One model	0.575643	1.151286	1	0.283280107	0.00995
Carsonella HT RpoC fix 0.2	17	-16441.70194	Two ratio model	4.99838	9.99676	1	0.001568159	
Carsonella HT RpoC fix 1	17	-16443.92514	Two ratio model	7.22158	14.44316	1	0.000144454	

Carsonella HT RpoC fix 1.2	17	-16444.0561	Two ratio model	-7.35254	14.70508	1	0.000125707	
Two ratio model Carsonella HT RpoC	18	-16436.70356	One model	0.248853	0.497706	1	0.480509824	0.01974
Carsonella PC RpoC fix 0.2	17	-16446.61749	Two ratio model	9.71671	19.43342	1	1.04168E-05	
Carsonella PC RpoC fix 1	17	-16449.77572	Two ratio model	12.87494	25.74988	1	3.88651E-07	
Carsonella PC RpoC fix 1.2	17	-16449.92826	Two ratio model	-13.02748	26.05496	1	3.31835E-07	
Two ratio model Carsonella PC RpoC	18	-16436.90078	One model	0.051633	0.103266	1	0.74794496	0.01661
Carsonella PV RpoC fix 0.2	17	-16434.4716	Two ratio model	2.01468	4.02936	1	0.044714901	
Carsonella PV RpoC fix 1	17	-16436.95123	Two ratio model	4.49431	8.98862	1	0.002716661	
Carsonella PV RpoC fix 1.2	17	-16437.1075	Two ratio model	4.65058	9.30116	1	0.002290088	
Two ratio model Carsonella PV RpoC	18	-16432.45692	One model	4.495493	8.990986	1	0.002713146	0.05077
Carsonella YCC RpoC fix 0.2	17	-16436.95509	Two ratio model	-0.00207	0.00414	1	0.948697249	
Carsonella YCC RpoC fix 1	17	-16436.95656	Two ratio model	-0.0006	0.0012	1	0.972365995	
Carsonella YCC RpoC fix 1.2	17	-16436.95665	Two ratio model	-0.00051	0.00102	1	0.974521942	
Two ratio model Carsonella YCC RpoC	18	-16436.95716	One model	-0.004747	0.009494	1	0.922379236	194.5297
One model Carsonella RpoD	17	-3901.869396	Free model	-6.623599	13.247198	14	0.507158048	
Free model Carsonella RpoD	31	-3895.245797						
Carsonella CE RpoD Fix 0.2	17	-3900.550845	Two ratio model	0.171147	0.342294	1	0.558508065	
Carsonella CE RpoD Fix 1	17	-3900.4158	Two ratio model	-0.036102	0.072204	1	0.78815438	
Carsonella CE RpoD Fix 1.2	17	-3900.409845	Two ratio model	0.030147	0.060294	1	0.806031864	
Two ratio model Carsonella CE RpoD	18	-3900.379698	One model	1.453596	2.907192	1	0.088185291	999
Carsonella-CS RpoDfix-0-2	17	-3901.698898	Two ratio model	0.184632	0.369264	1	0.543405725	
Carsonella-CS RpoDfix 1.0	17	-3901.807563	Two ratio model	0.293297	0.586594	1	0.443739228	
Carsonella-CS RpoDfix1-2	17	-3901.812781	Two ratio model	0.298515	0.59703	1	0.439713465	
Two ratio model Carsonella CS RpoD	18	-3901.514266	One model	-0.35513	0.71026	1	0.399357512	0.03863
Carsonella-DC RpoDfix 0-2	17	-3901.869446	Two ratio model	4.7E-05	9.4E-05	1	0.992264343	
Carsonella-DC RpoDfix 1.0	17	-3901.869479	Two ratio model	1.4E-05	2.8E-05	1	0.995778011	
Carsonella-DC RpoDfix1-2	17	-3901.869482	Two ratio model	-1.1E-05	2.2E-05	1	0.996257603	

Two ratio model Carsonella DC RpoD	18	-3901.869493	One model	9.7E-05	0.000194	1	0.988887113	123.39769
Carsonella HC RpoD Fix 0.2	17	-3902.076579	Two ratio model	0.581963	1.163926	1	0.280652808	
Carsonella-HC RpoDfix 1.0	17	-3902.647531	Two ratio model	1.152915	2.30583	1	0.128889416	
Carsonella-HC RpoDfix1-2	17	-3902.68332	Two ratio model	1.188704	2.377408	1	0.123101648	
Two ratio model Carsonella HC RpoD	18	-3901.494616	One model	-0.37478	0.74956	1	0.386615573	0.03096
Carsonella-HT RpoDfix 0-2	17	-3901.54634	Two ratio model	0.004478	0.008956	1	0.924603839	
Carsonella-HT RpoDfix 1.0	17	-3901.544956	Two ratio model	0.003094	0.006188	1	0.937300006	
Carsonella-HT RpoDfix1-2	17	-3901.545647	Two ratio model	0.003785	0.00757	1	0.930666944	
Two ratio model Carsonella HT RpoD	18	-3901.541862	One model	0.327534	0.655068	1	0.418306544	0.37157
Carsonella-PC RpoDfix 0-2	17	-3900.62097	Two ratio model	0.306846	0.613692	1	0.433401084	
Carsonella-PC RpoDfix 1.0	17	-3900.379684	Two ratio model	0.06556	0.13112	1	0.717273599	
Carsonella-PC RpoDfix1-2	17	-3900.368858	Two ratio model	0.054734	0.109468	1	0.740750625	
Two ratio model Carsonella PC RpoD	18	-3900.314124	One model	-1.555272	3.110544	1	0.077786975	999
Carsonella-PV RpoDfix 0-2	17	-3900.001136	Two ratio model	0.522521	1.045042	1	0.306651729	
Carsonella-PV RpoDfix 1.0	17	-3899.599521	Two ratio model	0.120906	0.241812	1	0.622900445	
Carsonella-PV RpoDfix1-2	17	-3899.579881	Two ratio model	0.101266	0.202532	1	0.652684804	
Two ratio model Carsonella PV RpoD	18	-3899.478615	One model	-2.390781	4.781562	1	0.028766017	999
Carsonella-YCCR RpoDfix 0-2	17	-3901.869446	Two ratio model	-4E-06	8E-06	1	0.997743245	
Carsonella-YCCR RpoDfix 1.0	17	-3901.86948	Two ratio model	3E-05	6E-05	1	0.993819675	
Carsonella-YCCR RpoDfix 1.2	17	-3901.869482	Two ratio model	3.2E-05	6.4E-05	1	0.993616992	
Two ratio model Carsonella YCCR RpoD	18	-3901.86945	One model	5.4E-05	0.000108	1	0.99170829	17.13565
Carsonella-BC RpoDfix 0-2	17	-3905.057412	Two ratio model	4.163994	8.327988	1	0.003903887	
Carsonella-BC RpoDfix 1.0	17	-3906.817874	Two ratio model	5.924456	11.848912	1	0.00057695	
Carsonella-BC RpoDfix1-2	17	-3906.97055	Two ratio model	6.077132	12.154264	1	0.000489758	
Two ratio model Carsonella BC RpoD	18	-3900.893418	One model	-0.975978	1.951956	1	0.162376229	0.00952

[a]The test branch model for each lineage in each subunit, where the value fixed for D_N/D_S value is indicated, [b]The parameters number, [c]Log-likelihood, [d]The model for comparison, [e]The difference between of the log-likelihood of the two compared models, [f]Twice the difference of log-likelihood between the models, [g]The freedom degree calculated by the difference between the numbers (np) of the compared models, [h]The LRT by the distribution of x^2 with the degree freedom calculated, [i]The D_N/D_S value estimated by the two ratio model for each subunit of each strain.
