# Supplementary material for: RNA polymerases in strict endosymbiont bacteria with extreme genome reduction show distinct erosions that might result in limited and differential promoter recognition
Source: PLoS One. 2021 Jul 29;16(7):e0239350. doi: 10.1371/journal.pone.0239350 (PMC8321222; doi:10.1371/journal.pone.0239350)
Supplement: S3 Table — (PDF) [file pone.0239350.s008.pdf]

Table S3. Results of selective pressure obtained by branch-site model

Subunit[a]	Foreground branch[b]	2ΔlnLc[c]	ω[d]	Proportion of sites class[e]	Positively selected sites[f]
**α subunit (RpoA)**	*H. cicadicola* Dsem	9.7 (0.00184252)	ω_0=0.18124<br>ω_1=1<br>ω_2=24.2456	0: 0.554<br>1: 0.157378<br>2a: 0.22998<br>2b:0.06402	6A(0.710), 9R(0.601), 10L(0.872), 14I(0.795), 22A(0.898), 24A(0.907), 25A(0.848), 27I(0.653), 30E(0.577), 35S(0.708), 43R(0.763), 45S(0.803), 50T(0.766), 53Q(0.934), 54L(0.624), 55V(0.971*), 77V(0.797), 94A(0.712), 95Q(0.974*), 98A(0.699), 102R(0.840), 103V(0.514), 105A(0.837), 110V(0.689), 115H(0.979*), 125S(0.930), 126S(0.878), 127D (0.508), 131R(0.829), 137Q(0.873), 142T(0.543)
	H. cicadicola Tetund2	18.892 (0.00001536)	ω_0=0.12<br>ω_1=1<br>ω_2=10.34	0: 0.546<br>1: 0.150<br>2a: 0.2383<br>2b:0.06554	1S(1.000**), 2A (0.946), 3V(0.677), 4E(0.527), 5L(0.826), 6K(0.642), 8G(0.525), 9R(0.679), 11G(0.583), 15Q(0.845), 17A(0.658), 21A(0.676), 23S(0.680), 33V(0.506), 41V(0.533), 43L(0.550), 46P(0.825), 49A(0.712), 50P(0.759), 56Y(0.982*), 57L(0.854), 58T(0.922), 59A(0.923), 60L(0.733), 61V(0.998**), 62C(0.943), 63L(0.772), 64I(0.805), 65R (0.716), 66V(0.987*), 67S(0.999**), 68E(0.988*), 69F(0.829), 70A(0.998**), 71T(0.986*), 76P(0.802), 79K(0.705), 84T(0.700), 89E(0.733), 90C(0.713), 101S(0.680), 103R(0.538), 116(0.835), 117V(0.630), 129H(0.771), 143D(0.573), 144L(0.999**), 145R(0.999**

[a]The RNAP subunits tested by the branch-site model, [b]The lineage tested as the foreground branch, [c] Twice the difference of log-likelihood between the models, the LRT by the χ^2 value obtained is indicated in parentheses [d] D_N/D_S values for the background lineage, [e] Sites class 0 and 1 comprise sites under purifying (0< ω0<1) and neutral selection (ω=1), in both foreground and background lineage. Site class 2 allows a proportion of positive sites in the foreground lineage where 2a includes sites under purifying selection (0< ω0<1) in the background lineage, 2b

includes the neutral sites in the background lineages. Both 2a and 2b allow a proportion of sites in the foreground lineage to be in positive selection (ω2=1) [f] Sites with elevated D_N/D_S values detected by the branch-site model. BEB posterior probabilities are shown in parentheses (*p-val0.95 and **p-val0.99).
