# Supplementary material for: RNA polymerases in strict endosymbiont bacteria with extreme genome reduction show distinct erosions that might result in limited and differential promoter recognition
Source: PLoS One. 2021 Jul 29;16(7):e0239350. doi: 10.1371/journal.pone.0239350 (PMC8321222; doi:10.1371/journal.pone.0239350)
Supplement: S4 Table — (PDF) [file pone.0239350.s009.pdf]

Table S4. Changes of free energy by in silico mutations in selected sites of α subunit homodimer predicted for *Hodgkinia* TETUND2

**Predicted dimer**[a]	**Mutation**[b]	**ΔΔG**[c]
Model 3	HB118P	1.701
Model 14	VA68S	1.001
	VA68N	0.874
	VA68Q	0.993
	YB58L	1.273
	YB58H	1.657
	AB61G	0.907
	AB61D	1.207
	VB63R	1.008
	VB63E	0.918
	HB118P	1.701
Model 15	VA63R	1.008
	VA63E	0.918
	HA118P	1.701
	HB118P	1.701

[a]Three-dimensional model predicted by ClusPro v.2.0 tool for the α subunit dimers of Hodgkinia TETUND2 [b] *In silico* substitutions in the residues under positive selection [c]ΔΔGwt->mut=ΔGmut-ΔGwt. A strongly favorable mutation has ΔΔG≤-1kcal/mol.
