# Supplementary material for: RNA polymerases in strict endosymbiont bacteria with extreme genome reduction show distinct erosions that might result in limited and differential promoter recognition
Source: PLoS One. 2021 Jul 29;16(7):e0239350. doi: 10.1371/journal.pone.0239350 (PMC8321222; doi:10.1371/journal.pone.0239350)
Supplement: S5 Table — (PDF) [file pone.0239350.s010.pdf]

Table S5. Bacteria with similar %GC to endosymbiotic bacteria with reduced genome

[a]Genome size in Megabase pairs, [b]The GC percent content in the genome.

Microrganism name	Lifestyle	Genome size[a]	GC%[b]
**Alpha proteobacterias**			
Methylobacterium sp. 4-46	Free-living	7.73703	71.52
Erythrobacter atlanticus	Free-living	3.22636	58.35
Ehrlichia ruminantium str. Welgevonden	Free-living	1.51635	27.5
Wolbachia endosymbiont of Onchocerca ochengi	Endosymbiont	0.95799	32.1
Neorickettsia sennetsu str. Miyayama	Endosymbiont	0.859006	41.1
**Beta proteobacteria**			
Rubrivivax gelatinosus IL144	Free-living	5.04325	71.2
C. Symbiobacter mobilis CR	Endosymbiont	2.99184	59.1
C. Profftella armatura DC	Endosymbiont	0.464857	24.2
C. Zinderia insecticola CARI	Endosymbiont	0.208564	13.5
**Gamma proteobacteria**			
Xanthomonas sacchari	Free-living	5.00611	68.96
Haemophilus influenzae PittEE	Free-living	1.81303	38
Serratia symbiotica	Free-living	1.76276	29.2
Buchnera aphidicola BCc	Endosymbiont	0.422434	20.2

[a]Genome size in Megabase pairs, [b]The GC percent content in the genome.
